# Mitochondrial *Zea mays* Brittle1-1 Is a Major Determinant of the Metabolic Fate of Incoming Sucrose and Mitochondrial Function in Developing Maize Endosperms

**DOI:** 10.3389/fpls.2019.00242

**Published:** 2019-03-12

**Authors:** Abdellatif Bahaji, Francisco José Muñoz, Jose María Seguí-Simarro, Carolina Camacho-Fernández, Alba Rivas-Sendra, Verónica Parra-Vega, Miroslav Ovecka, Jun Li, Ángela María Sánchez-López, Goizeder Almagro, Edurne Baroja-Fernández, Javier Pozueta-Romero

**Affiliations:** ^1^Instituto de Agrobiotecnología, Consejo Superior de Investigaciones Científicas, Gobierno de Navarra, Navarra, Spain; ^2^COMAV - Institute for Conservation & Improvement of Valencian Agrodiversity, Universitat Politècnica de València, Valencia, Spain; ^3^Department of Cell Biology, Faculty of Science, Centre of the Region Haná for Biotechnological and Agricultural Research, Palacky University, Olomouc, Czechia; ^4^College of Agronomy and Plant Protection, Qingdao Agricultural University, Qingdao, China

**Keywords:** ADPglucose, dual targeting, mitochondrial carrier family, mitochondrial retrograde signaling, starch, sucrose synthase, *Zea mays*

## Abstract

*Zea mays* Brittle1-1 (ZmBT1-1) is an essential component of the starch biosynthetic machinery in maize endosperms, enabling ADPglucose transport from cytosol to amyloplast in exchange for AMP or ADP. Although ZmBT1-1 has been long considered to be an amyloplast-specific marker, evidence has been provided that ZmBT1-1 is dually localized to plastids and mitochondria (Bahaji et al., 2011b). The mitochondrial localization of ZmBT1-1 suggested that this protein may have as-yet unidentified function(s). To understand the mitochondrial ZmBT1-1 function(s), we produced and characterized transgenic *Zmbt1-1* plants expressing ZmBT1-1 delivered specifically to mitochondria. Metabolic and differential proteomic analyses showed down-regulation of sucrose synthase (SuSy)-mediated channeling of sucrose into starch metabolism, and up-regulation of the conversion of sucrose breakdown products generated by cell wall invertase (CWI) into ethanol and alanine, in *Zmbt1-1* endosperms compared to wild-type. Electron microscopic analyses of *Zmbt1-1* endosperm cells showed gross alterations in the mitochondrial ultrastructure. Notably, the protein expression pattern, metabolic profile, and aberrant mitochondrial ultrastructure of *Zmbt1-1* endosperms were rescued by delivering ZmBT1-1 specifically to mitochondria. Results presented here provide evidence that the reduced starch content in *Zmbt1-1* endosperms is at least partly due to (i) mitochondrial dysfunction, (ii) enhanced CWI-mediated channeling of sucrose into ethanol and alanine metabolism, and (iii) reduced SuSy-mediated channeling of sucrose into starch metabolism due to the lack of mitochondrial ZmBT1-1. Our results also strongly indicate that (a) mitochondrial ZmBT1-1 is an important determinant of the metabolic fate of sucrose entering the endosperm cells, and (b) plastidic ZmBT1-1 is not the sole ADPglucose transporter in maize endosperm amyloplasts. The possible involvement of mitochondrial ZmBT1-1 in exchange between intramitochondrial AMP and cytosolic ADP is discussed.

## Introduction

Mitochondria are the main sites of cellular respiration and ATP supply. They also play important roles in diverse processes such as redox homeostasis and provision of molecules that act as metabolic intermediates in essential biosynthetic pathways or as specific signals that modulate nuclear-encoded protein expression (Chandel, 2014). To connect internal metabolism with that of the surrounding cell, mitochondria possess solute carrier proteins in the inner membrane which, due to their common basic structure, are classed as members of the mitochondrial carrier family (MCF) (Haferkamp and Schmitz-Esser, 2012; Taylor, 2017). In plants, MCF proteins are involved in the transport of nucleotides, phosphate, di- and tri-carboxylates, amino acids, and cofactors across the mitochondrial membrane. They are all presumed to be targeted to the mitochondrial inner membrane, although some of them have been shown to occur in peroxisomes, glyoxysomes, plasma membrane, and plastids (Sullivan and Kaneko, 1995; Fukao et al., 2001; Bedhomme et al., 2005; Palmieri et al., 2006; Bahaji et al., 2011a,b).

Brittle1 (BT1) proteins are members of the MCF that occur only in plants. At the transcriptional level, maize plants express two *BT1* homologs: Zm*BT1-1* and Zm*BT1-2* (Kirchberger et al., 2007). Whereas Zm*BT1-2* shows a ubiquitous expression pattern in heterotrophic and autotrophic tissues, Zm*BT1-1* expression is developmentally regulated, being high in maize endosperms 12–25 days after pollination (DAP) and undetectable in non-endosperm tissues and suspension cultures (Sullivan et al., 1991; Cao et al., 1995; Sullivan and Kaneko, 1995; Cao and Shannon, 1996; Kirchberger et al., 2007). Zm*BT1-1* encodes a protein with a predicted molecular mass of ca. 47 kDa (Sullivan et al., 1991). In maize endosperms, ZmBT1-1 is present as three 39, 40, and 44 kDa proteins (Cao et al., 1995; Sullivan and Kaneko, 1995), the former two being processing products generated within the plastidial compartment (Li et al., 1992).

The *bt1-1* locus of maize was identified in 1926 by mutations that severely decreased the amount of starch deposition in the endosperm and resulted in seeds with a collapsed angular appearance at maturity that germinated slowly and produced plants of low vigor (Mangelsdorf, 1926). *Zmbt1-1* endosperms accumulate high levels of the starch precursor molecule, ADPglucose (Shannon et al., 1996). Import studies using amyloplasts isolated from maize endosperms showed that amyloplasts can transport ADPglucose in counter-exchange with AMP and ADP (Shannon et al., 1998). These studies also showed that the incorporation of externally applied ADPglucose into starch in *Zmbt1-1* amyloplasts was reduced to about 25% compared with wild type (WT) amyloplasts (Shannon et al., 1998). Overall, the data indicated that ZmBT1-1 is an essential component of the starch biosynthetic machinery in maize endosperms, enabling the transport into the amyloplast of cytosolic ADPglucose produced by ADPglucose pyrophosphorylase (AGP) and sucrose synthase (SuSy) (Bahaji et al., 2014; Boehlein et al., 2018) in exchange with ADP produced by starch synthase as schematically illustrated in Supplemental Figure 1 (Kleczkowski, 1996) (Shannon et al., 1996, 1998).

Although ZmBT1-1 has been long considered to be an amyloplast-specific marker (Sullivan and Kaneko, 1995; Shannon et al., 1998; Kirchberger et al., 2007), confocal fluorescence microscopy studies using plants stably expressing GFP fusions of ZmBT1-1, and electron microscopic immunocytochemical analyses of maize endosperms, have provided evidence that ZmBT1-1 is dually localized to plastids and mitochondria (Bahaji et al., 2011b). These studies also showed that ZmBT1-1 N-terminal extensions comprise targeting sequences recognized exclusively by the plastidial compartment, whereas sequences targeting to mitochondria are localized within the mature part of ZmBT1-1. The mitochondrial localization of ZmBT1-1 suggested that this protein may have as-yet unidentified function(s). To get insights into the role(s) of mitochondrial ZmBT1-1, in this work we conducted proteomic, metabolic, and microscopic characterization of developing endosperms of *Zmbt1-1* plants and transgenic *Zmbt1-1* plants expressing ZmBT1-1 delivered specifically to mitochondria. Our findings show that mitochondrial ZmBT1-1 is a decisive factor in primary metabolism and mitochondrial function in developing maize endosperms and raise important questions regarding the role of BT1 in cereal endosperms.

## Materials And Methods

### Plants, Growth Conditions, and Sampling

The work was performed using WT plants (the hybrid W23/M14/W64A), a *Zmbt1-1* mutant in a W23/M14/W64A background provided by the Maize Genetics COOP Stock Center (*bt1-m1::dSpm*, ref. 514N), which contains a ca. 3.3 kbp defective *Suppressor-mutator* (*dSpm*) in the third exon of *ZmBT1-1* (Sullivan et al., 1991) (Supplemental Figure 2A) and a *Sh2* mutant in a OH43 background provided by the Maize Genetics COOP Stock Center (*sh2/OH43*, ref. 333D). The indentity of the *Zmbt1-1* mutant was confirmed by PCR using the O1 (5′-CGAGACGCTGAAGCGGCTCTAC-3′) and O2 (5′-CACGATCCGGAAACACCACATC-3′) *ZmBT1-1* specific primers (the latter hybridizes with a genomic sequence occurring downstream of the *ZmBT1-1* stop codon, Supplemental Figures 2A,B) as well as the *dSpm*-specific O3 primer (5′-GGACTTGAACTTGTATGAATATTG-3′) (Supplemental Figures 2A,B). We also used *Zmbt1-1* plants transformed with *UBI-ZmBT1-1, UBI-*Δ*TP-ZmBT1-1*, and *UBI-MitTPr-*Δ*TP-ZmBT1-1* (designated *Zmbt1-1::ZmBT1-1, Zmbt1-1::*Δ*TP-ZmBT1-1, Zmbt1-1::MitTPr-*Δ*TP-ZmBT1-1*, respectively) that were generated in two steps:

***Step one:*** Production of HiII plants (Armstrong et al., 1991) transformed with *UBI-ZmBT1-1, UBI-*Δ*TP-ZmBT1-1* and *UBI-MitTPr-*Δ*TP-ZmBT1-1*. To achieve this, HiII immature embryo-derived callus cultures were transformed using the biolistic gun-mediated method (Wang and Frame, 2009) and the pAHC25-ZmBT1-1, pAHC25-ΔTP-ZmBT1-1 and pAHC25-MiTPr-ΔTP-ZmBT1-1 plasmids (Supplemental Figure 3). These plasmids were produced from the pAHC25 plasmid (Christensen and Quail, 1996) by Gateway technology (Invitrogen), and their identities confirmed by sequencing. They contain, respectively, the *ZmBT1-1*, Δ*TP-ZmBT1-1*, and *MitTPr-*Δ*TP-ZmBT1-1* genes, each under the transcriptional control of the *Ubi-1* promoter, and a selectable (*bar*) gene (Supplemental Figure 3). Plantlets regenerated in medium containing the herbicide glufosinate were transplanted into pots, and further selected by spraying with the herbicide glufosinate (0.1%). Herbicide selection and PCR analyses (see below) were conducted for every generation, and the herbicide-resistant and PCR-positive plants were self-pollinated until homozygous transgenic HiII lines were generated. PCR-confirmation of the integration of *UBI-ZmBT1-1, UBI-*Δ*TP-ZmBT1-1*, and *UBI-MitTPr-*Δ*TP-ZmBT1-1* into the plant genome was conducted using the *Ubi-1* promoter-specific O4 primer (5′-GCATATGCAGCAGCTATATGTG-3′) and the *ZmBT1-1-*specific O5 primer (5′-GGTGCGGGTTGGCGATCTTG-3′) (Supplemental Figures 2C,D). Transformation with *UBI-*Δ*TP-ZmBT1-1* was further confirmed by PCR using O4 and the O6 primer (5′-GGGACCTGCAATGACGACCA-3′) specific for the ZmBT1-1 TP encoding sequence (Supplemental Figures 2C,E). Transformation with *UBI-MitTPr-*Δ*TP-ZmBT1-1* was further PCR-confirmed using O5 and the O7 primer (5′-ATGGCTATGGCTGTTTTCCGC-3′) specific for the MitTPr encoding sequence (Supplemental Figures 2C,F). All the transformations were further confirmed by sequencing of the amplicons obtained by PCR.

***Step two***: Crossing *Zmbt1-1* plants with the transgenic HiII plants generated as above. The seeds obtained were germinated and plants selected by spraying with glufosinate. Herbicide selection and PCR analyses were conducted, and herbicide-resistant and PCR-positive plants were self-pollinated, backcrossed to *Zmbt1-1* for a total of three generations, and self-pollinated until homozygous *Zmbt1-1::ZmBT1-1, Zmbt1-1::*Δ*TP-ZmBT1-1*, and *Zmbt1-1::MitTPr-*Δ*TP-ZmBT1-1* plants were generated.

For subcellular localization of GFP-tagged proteins, we produced HiII plants transformed with *UBI-*Δ*TP-ZmBT1-1-GFP, UBI-MiTPr-GFP*, and *UBI-MitTPr-*Δ*TP-ZmBT1-1-GFP* (designated as Δ*TP-ZmBT1-1-GFP, MiTPr-GFP*, and *MitTPr-*Δ*TP-ZmBT1-1-GFP*, respectively) using the pAHC25-ΔTP-ZmBT1-1-GFP, pAHC25-MiTPr-GFP, and pAHC25MiTPr-ΔTP-ZmBT1-1-GFP plasmids (Supplemental Figure 3).

Ten plants per line were grown in 35 L pots in greenhouse conditions. For biochemical and proteomic analyses, seeds were collected at the indicated developmental stage, and endosperms immediately extracted, freeze-clamped, and ground to a fine powder in liquid nitrogen with a pestle and mortar.

### Analytical Procedures

For measurement of glucose-6-phosphate (G6P), fructose-6-phosphate, glucose-1-phosphate, UDPglucose and ADPglucose, a 0.5 g aliquot of the frozen powdered endosperm (see above) was resuspended in 1 ml of 1 M HClO_4_, left at 4°C for 2 h and centrifuged at 10,000 × g for 5 min. The supernatant was neutralized with K_2_CO_3_ and centrifuged at 10,000 × g. G6P, fructose-6-phosphate, and glucose-1-phosphate in supernatants were determined by HPLC with pulsed amperometric detection on a DX-500 Dionex system with a CarboPac 10 column according to the gradient separation application method suggested by the supplier (100 mM NaOH/100 mM sodium acetate to 100 mM NaOH/500 mM sodium acetate over 40 min). UDPglucose and ADPglucose were measured as described in Li et al. (2013), by HPLC on a system obtained from Waters Associates fitted with a Partisil-10-SAX column. Alanine and GABA contents were measured as described by Loiret et al. (2009). Ethanol was measured as described by Licausi et al. (2010). Recovery experiments were carried out by adding known amounts of metabolite standards to the frozen tissue slurry immediately after the addition of extraction solutions. The difference between the measurements from samples with and without added standards was used as an estimate of the percentage of recovery. All data were corrected for losses during extraction. Starch from 24 DAP endosperms and dry seeds was measured with an amyloglucosidase–based test kit (Boehringer Mannheim, Germany).

### Assays for Total Invertase and Sucrose Synthase Activites

One g of the frozen powder (see above) was resuspended at 4°C in 5 ml of 100 mM HEPES (pH 7.5), 2 mM EDTA, and 5 mM dithiothreitol (extraction buffer). The suspension was desalted, resuspended in 5 ml extraction buffer, and assayed for enzymatic activities. We verified that this procedure did not result in loss of enzymatic activity by comparing activity in extracts prepared from the frozen powder with extracts prepared by homogenizing fresh tissue in extraction medium. Total invertase and SuSy activities were measured as described by Baroja-Fernández et al. (2009). One unit (U) is defined as the amount of enzyme that catalyzes the production of 1 μmol of product per min.

### Western Blot Analyses

For immunoblot analyses, 100 mg of the fine frozen endosperm powder was resuspended in 300 μl of 50 mM Hepes pH 7.0, 2 mM EDTA, and 10 mM dithiothreitol, incubated 30 min at 4°C, and centrifuged at 10,000 × g for 15 min. Proteins occurring in the supernatant were then separated on 15% SDS-PAGE, transferred to PVDF filters, and immunodetected using the antisera raised against recombinant ZmBT1-1 (Bahaji et al., 2011a), and a goat anti-rabbit IgG alkaline phosphatase conjugate as secondary antibody (Sigma).

### Iodine Starch Staining

Thirty DAP seeds were stained in iodine solution [KI 2% (w/v), I_2_ 1% (w/v)] for 30 min, rinsed briefly in deionized water and photographed.

### Isobaric Labeling-Based Differential Proteomic Analyses

These analyses were conducted essentially as decribed in Sánchez-López et al. (2016) for Arabidopsis leaves but with the following modifications. For protein sample preparation samples were prepared by grinding 200 mg of endosperm material from 24 DAP developing seeds into a fine powder under liquid nitrogen using a pre-cooled mortar and pestle. For the data analyses, MS/MS spectra were exported to MGF format using Peak View v1.2.0.3 (Sciex, Redwood City, CA) and searched using Mascot Server 2.5.1, OMSSA 2.1.9, X!TANDEM 2013.02.01.1 and MyriMatch 2.2.140 against a composite target/decoy database for maize built from the 85,535 sequences from UniProt Knowledgebase, together with commonly occurring contaminants. The cut-off for differentially regulated proteins was set at a FDR ≤ 5%. Functional characterization of the differentially expessed proteins was performed using the MapMan tool (https://mapman.gabipd.org/) (Thimm et al., 2004).

### Confocal Microscopy

Subcellular localization of GFP in Δ*TP-ZmBT1-1-GFP, MiTPr-GFP*, and *MitTPr-*Δ*TP-ZmBT1-1-GFP* plants was performed using D-Eclipse C1 confocal microscope (Nikon, Japan) equipped with standard Ar 488 laser excitation, BA515/30 filter for green emission, and a BA650LP filter for red emission.

### Transmission Electron Microscopy

We characterized samples from leaves and 24 DAP endosperms of WT, *Zmbt1-1, Sh2*, and *Zmbt1-1::MitTPr-*Δ*TP-ZmBT1-1* plants. Once excised, samples were immediately transferred to aluminum sample holders, cryoprotected with 150 mM sucrose, frozen in a Leica electron microscopic HPM-100 high-pressure freezer (Leica Microsystems, Vienna), transferred to liquid nitrogen, and processed according to Seguí-Simarro (2015). In brief, samples were freeze substituted with anhydrous acetone +2% OsO_4_ at −80°C for 4 days, followed by slow warming to room temperature over a period of 24 h. After rinsing several times in acetone, they were removed from the holders and embedded with increasing concentrations of Spurr resin in acetone according to the following schedule: 3 h in 2% resin, 3 h in 5% resin, 15 h in 10% resin, 8 h in 25% resin, 15 h in 50% resin, 8 h in 75% resin, and 40 h in 100% resin. Resin polymerization was performed at 70°C for 30 h. Ultrathin (~80 nm) sections were then obtained using a Leica UC6 ultramicrotome, mounted on formvar-coated, 150 mesh copper grids, stained with 2% uranyl acetate for 6 min and with lead citrate prepared as described in Reynolds (1963) for 4 min, and observed in a Jeol JEM 1010 transmission electron microscope.

### Statistical Analysis

The data are presented as the means of four independent experiments, with 3–5 replicates for each experiment (means ± SE). The significance of differences between WT, *Zmbt1-1::ZmBT1-1, Zmbt1-1::*Δ*TP-ZmBT1-1*, and *Zmbt1-1::MitTPr-*Δ*TP-ZmBT1-1* endosperms was statistically evaluated by Student's *t*-test using the SPSS software. Differences were considered significant at a probability level of *P* < 0.05.

## Results

### Generation of Transgenic *Zmbt1-1* Plants Expressing ZmBT1-1 Delivered Specifically to Mitochondria

We generated transgenic homozygous *Zmbt1-1* maize plants transformed with either *UBI-ZmBT1-1* or *UBI-*Δ*TP-ZmBT1-1* as described in Methods. These plants express, respectively, *ZmBT1-1* and Δ*TP-ZmBT1-1* under the control of the *Ubi-1* promoter; the latter construct encodes a ca. 44 kDa truncated form of ZmBT1-1 (designated as ΔTP-ZmBT1-1) that lacks 24 amino acids from the N-terminal extension which potentially acts as a plastidial transit peptide (TP). We also generated *Zmbt1-1* plants transformed with *UBI-MitTPr-*Δ*TP-ZmBT1-1*, which express a mitochondria-targeting pre-sequence [the N-terminus of the F1-ATPase γ-subunit (Niwa et al., 1999)] fused to ΔTP-ZmBT1-1. The identities of the *Zmbt1-1* mutant, and the homozygous *Zmbt1-1* maize plants transformed with *UBI-ZmBT1-1, UBI-*Δ*TP-ZmBT1-1*, or *UBI-MitTPr-*Δ*TP-ZmBT1-1* (designated as *Zmbt1-1::ZmBT1-1, Zmbt1-1::*Δ*TP-ZmBT1-1*, and *Zmbt1-1::MitTPr-*Δ*TP-ZmBT1-1*, respectively) were confirmed by PCR and sequencing of the amplicons obtained (Supplemental Figure 2).

**Figure 1:**
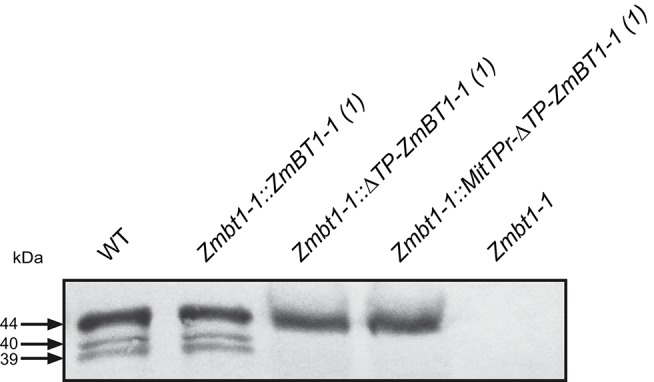
Western blot analysis of ZmBT1-1 in proteins extracted from 24 DAP WT (W23/M14/W64A), *Zmbt1-1::ZmBT1-1, Zmbt1-1::*Δ*TP-ZmBT1-1, Zmbt1-1::MitTPr-*Δ*TP-ZmBT1-1*, and *Zmbt1-1* endosperms. The gel was loaded with 30 μg protein per lane.

Western blot analyses of ZmBT1-1 revealed bands of ca. 39, 40, and 44 kDa in WT endosperms (Figure 1), which is consistent with previous reports (Cao et al., 1995; Sullivan and Kaneko, 1995). As expected, no polypeptides cross-reacting with the ZmBT1-1 antisera could be detected in *Zmbt1-1* endosperms (Figure 1). Similar to WT endosperms, *Zmbt1-1::ZmBT1-1* endosperms expressed three proteins of ca. 39, 40, and 44 kDa that cross-reacted with ZmBT1-1 antisera (Figure 1). In contrast, *Zmbt1-1::*Δ*TP-ZmBT1-1* and *Zmbt1-1::MitTPr-*Δ*TP-ZmBT1-1* endosperms accumulated a single ca. 44 kDa protein that cross-reacted with the ZmBT1-1 antisera (Figure 1). No plastidial ZmBT1-1 processing product (e.g., 39 and 40 kDa proteins; Li et al., 1992) could be detected in *Zmbt1-1::*Δ*TP-ZmBT1-1* and *Zmbt1-1::MitTPr-*Δ*TP-ZmBT1-1* endosperms (Figure 1).

TargetP (http://www.cbs.dtu.dk/services/TargetP/) (Emanuelsson et al., 2000) predicts mitochondrial localization for both ΔTP-ZmBT1-1 and MitTPr-ΔTP-ZmBT1-1. To confirm that ΔTP-ZmBT1-1 was specifically targeted to mitochondria in *Zmbt1-1::*Δ*TP-ZmBT1-1* and *Zmbt1-1::MitTPr-*Δ*TP-ZmBT1-1* endosperms we carried out confocal fluorescence microscopy analyses of WT maize plants expressing Δ*TP-ZmBT1-1-GFP* and *MitTPr-*Δ*TP-ZmBT1-1-GFP*. We also analyzed fluorescence distribution in plants expressing the mitochondrial-targeting pre-sequence MitTPr fused with GFP (MitTPr-GFP). These analyses revealed that the GFP fluorescence distribution and motility patterns in Δ*TP-ZmBT1-1-GFP-* and *MitTPr-*Δ*TP-ZmBT1-1-GFP-* expressing plants were identical to those of plants expressing the mitochondrial marker MitTPr-GFP (see movies in Supplemental Videos 1–3), which is consistent with previous studies using Δ*TP-ZmBT1-1-GFP-* and *MitTPr-*Δ*TP-ZmBT1-1-GFP-* expressing Arabidopsis plants (cf. Figure 4, Bahaji et al., 2011b). These findings confirm that ΔTP-ZmBT1-1-GFP has a mitochondrial localization in Δ*TP-ZmBT1-1-GFP-* and *MitTPr-*Δ*TP-ZmBT1-1-GFP-* expressing maize plants.

### Delivery of ZmBT1-1 Specifically to Mitochondria Complements the Low Starch Content Phenotype of *Zmbt1-1* Seeds

**Figure 2:**
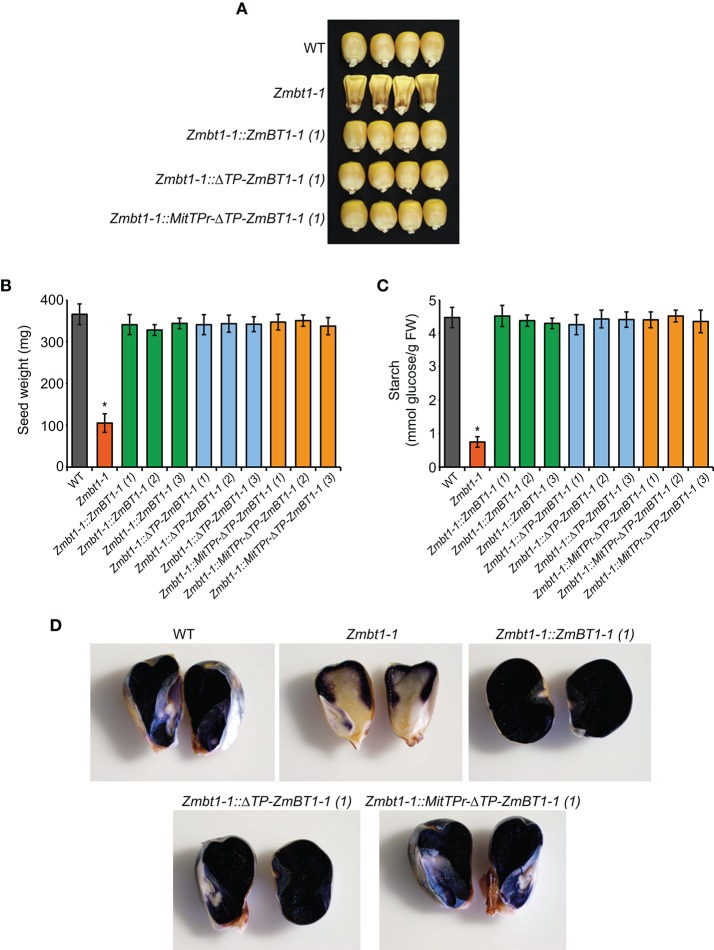
Delivery of ZmBT1-1 specifically to mitochondria complements the collapsed angular appearance and the low endosperm starch content of *Zmbt1-1* seeds. External appearance **(A)**, weight **(B)**, and starch content **(C)** of WT, *Zmbt1-1, Zmbt1-1::ZmBT1-1, Zmbt1-1::*Δ*TP-ZmBT1-1*, and *Zmbt1-1::MitTPr-*Δ*TP-ZmBT1-1* dry seeds. **(D)** Iodine staining of 30 DAP WT, *Zmbt1-1, Zmbt1-1::ZmBT1-1, Zmbt1-1::*Δ*TP-ZmBT1-1*, and *Zmbt1-1::MitTPr-*Δ*TP-ZmBT1-1* seeds. In **(B,C)**, values represent the mean ± SD of determinations on four independent samples from three independent lines each of *Zmbt1-1::ZmBT1-1, Zmbt1-1::*Δ*TP-ZmBT1-1*, and *Zmbt1-1::MitTPr-*Δ*TP-ZmBT1-1*. Asterisks indicate significant differences based on Student's *t*-tests. (*P* < 0.05, *Zmbt1-1* mutants vs. WT).

*Zmbt1-1* seeds showed a collapsed, angular appearance at maturity (Figure 2A) and were of reduced weight (Figure 2B) and starch content (Figure 2C), a finding which is consistent with previous reports (Mangelsdorf, 1926; Sullivan et al., 1991). As expected, these phenotypes could be reverted to WT by ectopic expression of *ZmBT1-1* (Figure 2). Notably, *Zmbt1-1::*Δ*TP-ZmBT1-1* and *Zmbt1-1::MitTPr-*Δ*TP-ZmBT1-1* seeds displayed a normal external appearance (Figure 2A), and their weights and starch contents were like those of WT seeds (Figures 2B,C). Iodine staining for starch localization in excised 30 DAP seeds showed an even distribution of starch in WT, *Zmbt1-1::ZmBT1-1, Zmbt1-1::*Δ*TP-ZmBT1-1*, and *Zmbt1-1::MitTPr-*Δ*TP-ZmBT1-1* endosperms, whereas in *Zmbt1-1* seeds starch accumulated exclusively in the upper part of the endosperm (Figure 2D).

*Zmbt1-1* seeds germinated slowly when compared with WT seeds (Supplemental Figure 4A), which is consistent with the findings of Mangelsdorf (1926). Slow germination resulted in delayed growth of *Zmbt1-1* plants when compared with WT plants of various genetic backgrounds (Supplemental Figure 4B). As expected, the slow germination and delayed growth phenotype of *Zmbt1-1* plants could be complemented by the ectopic expression of *ZmBT1-1* (Supplemental Figure 4)*. Zmbt1-1::*Δ*TP-ZmBT1-1* and *Zmbt1-1::MitTPr-*Δ*TP-ZmBT1-1* seeds displayed WT germination and growth phenotypes (Supplemental Figure 4).

### Delivery of ZmBT1-1 Specifically to Mitochondria Restores the High ADPglucose Content of Developing *Zmbt1-1* Endosperms to Wild Type Values

**Figure 3:**
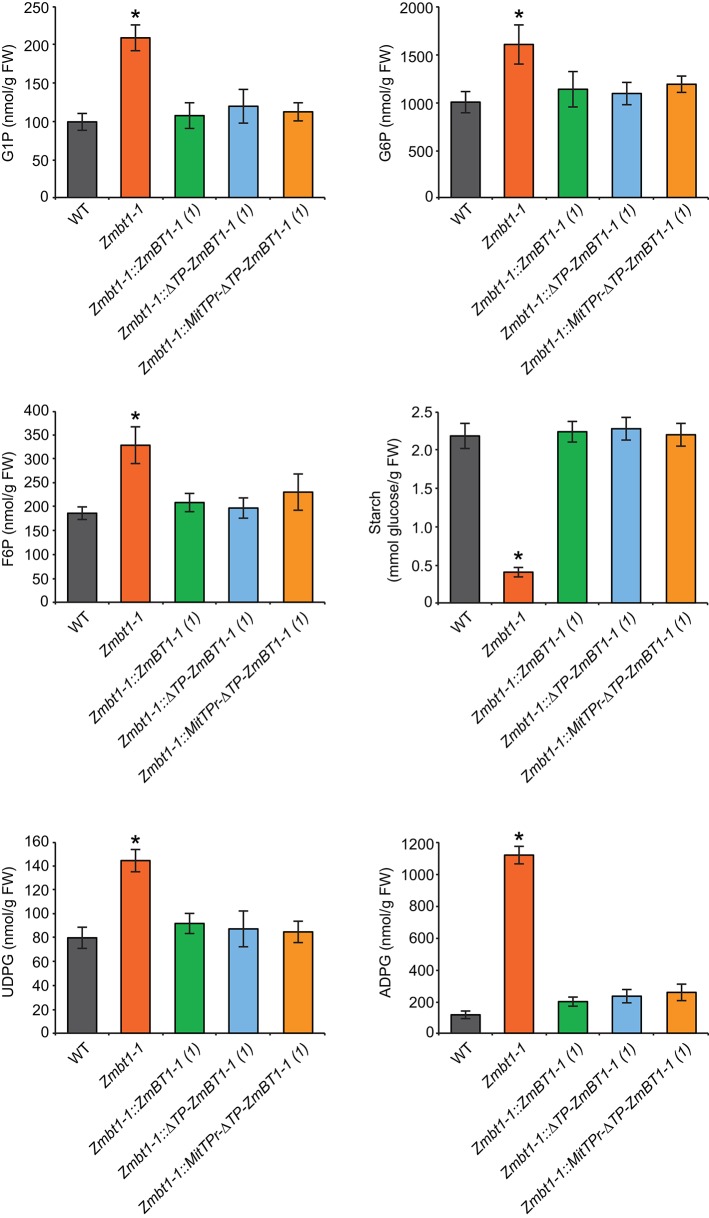
Delivery of ZmBT1-1 specifically to mitochondria reverts to WT the content of ADPglucose and other sucrose-to-starch biosynthetic intermediates in developing *Zmbt1-1* endosperms. The graphics represent the amounts of the metabolites indicated in 24 DAP endosperms from WT, *Zmbt1-1, Zmbt1-1::ZmBT1-1(1), Zmbt1-1::*Δ*TP-ZmBT1-1(1)*, and *Zmbt1-1::MitTPr-*Δ*TP-ZmBT1-1(1)* seeds. Values represent the mean ± SD of determinations on four independent samples. Asterisks indicate significant differences based on Student's *t*-tests. (*P* < 0.05, *Zmbt1-1* mutants vs. WT).

WT, *Zmbt1-1, Zmbt1-1::ZmBT1-1, Zmbt1-1::*Δ*TP-ZmBT1-1*, and *Zmbt1-1::MitTPr-*Δ*TP-ZmBT1-1* developing (24 DAP) endosperms were analyzed for contents of metabolic intermediates of the sucrose-to-starch conversion process. As expected, the starch content of *Zmbt1-1* endosperms was lower than that of WT endosperms. Levels of hexose-phosphates (e.g., fructose-6-phosphate, G6P and glucose-1-phosphate) in *Zmbt1-1* endosperms were higher than those in WT endosperms. UDPglucose and ADPglucose contents of *Zmbt1-1* endosperms were ca. 2- and 12-fold higher, respectively, than those of WT endosperms (Figure 3), an observation which is consistent with Shannon et al. (1996). Notably, the low starch content, and the high hexose-phosphate and nucleotide-sugar contents of developing *Zmbt1-1* endosperms could be reverted to WT levels not only by ectopic expression of *ZmBT1-1*, but also through expression of Δ*TP-ZmBT1-1* and *MitTPr-*Δ*TP-ZmBT1-1* (Figure 3).

### Knocking Out *ZmBT1-1* Promotes Changes in the Proteome of Maize Endosperms, Some of Which Can Be Reverted by the Delivery of ZmBT1-1 Specifically to Mitochondria

The results presented above provided evidence that mitochondrial ZmBT1-1 plays an important role in the sucrose-to-starch conversion process in maize endosperms. To obtain insights into the mechanisms influenced by the mitochondrial ZmBT1-1, we carried out high-throughput, isobaric labeling-based differential proteomic analyses of 24 DAP *Zmbt1-1* and WT endosperms, and 24 DAP *Zmbt1-1* and *Zmbt1-1::MitTPr-*Δ*TP-ZmBT1-1* endosperms.

#### Differential Proteomic Analysis of *Zmbt1-1* and WT Endosperms

**Figure 4:**
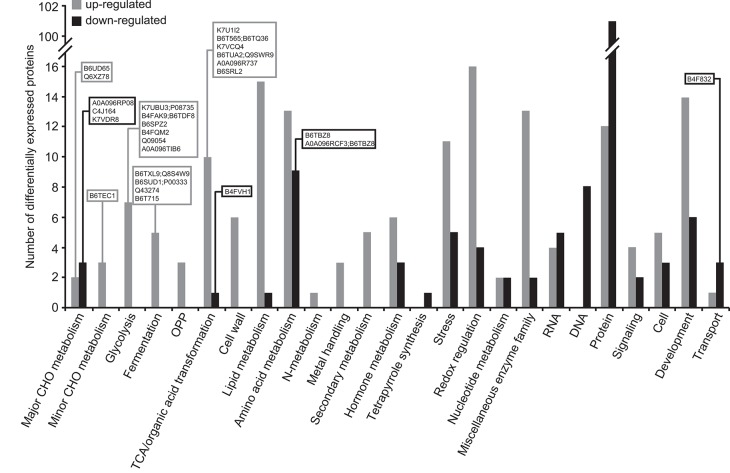
Functional categorization of the proteins differentially expressed in endosperms of *Zmbt1-1* and WT seeds. The number of proteins that are up- and down-regulated in *Zmbt1-1* endosperms is indicated by gray and black bars, respectively. Proteins are indicated by their accession numbers. Their descriptions are shown in Supplemental Table 1. Proteins discussed here are boxed.

As shown in Supplemental Tables 1, 2, 414 out of the 2183 proteins identified were differentially expressed in *Zmbt1-1* and WT endosperms, 35 of them being annotated as “uncharacterized proteins.” Among the population of 379 differentially expressed proteins (DEPs) with known functions, 191 were up-regulated, and 188 were down-regulated in *Zmbt1-1* (Supplemental Table 1). By using the broad classifications outlined by MapMan, the 379 proteins with known functions that were differentially expressed in *Zmbt1-1* and WT endosperms were assembled into 25 functional groups (Figure 4). The absence of ZmBT1-1 resulted in the down-regulation of the expression of several soluble starch synthase (SSS) isoforms and the major endosperm SuSy isoform, SH1 (Supplemental Table 1, Figure 4), and up-regulation of the expression of the major endosperm invertase isoform, the cell wall invertase 2 (CWI-2), sorbitol dehydrogenase (SDH), fructokinase (FK), glycolytic enzymes, and enzymes of the ethanolic fermentation pathway and the TCA cycle (Supplemental Table 1, Figure 4).

#### Differential Proteomic Analysis of *Zmbt1-1* and *Zmbt1-1::MitTPr-ΔTP-ZmBT1-1* Endosperms

One hundred and twenty-five out of the 2,152 proteins identified were differentially expressed in *Zmbt1-1 and Zmbt1-1::MitTPr-*Δ*TP-ZmBT1-1* endosperms, 7 of them being annotated as “uncharacterized proteins” (Supplemental Tables 3, 4). Among the population of 118 DEPs with known functions, 60 were up-regulated, and 58 were down-regulated in *Zmbt1-1* endosperms. When comparing the DEPs in *Zmbt1-1* and *Zmbt1-1::MitTPr-*Δ*TP-ZmBT1-1* endosperms (Supplemental Table 3) with those of *Zmbt1-1* and WT endosperms (Supplemental Table 1) we found that 53% of the proteins that were differentially expressed in the *Zmbt1-1* and *Zmbt1-1::MitTPr-*Δ*TP-ZmBT1-1* endosperms were also differentially expressed in the *Zmbt1-1* and WT endosperms (Supplemental Table 3). This indicates that the absence of mitochondrial ZmBT1-1 largely accounts for the differences in protein expression observed in *ZmBT1-1* and WT endosperms.

**Figure 5:**
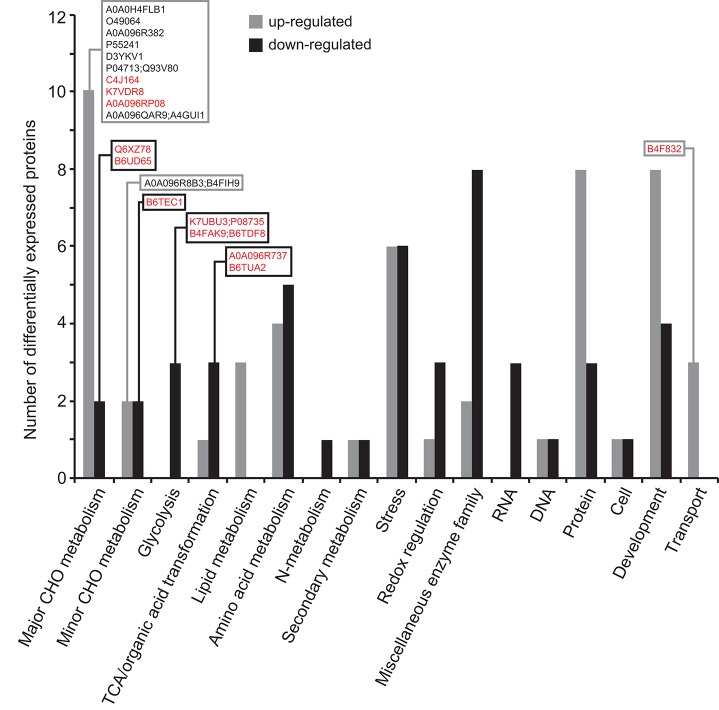
Functional categorization of the proteins differentially expressed in endosperms of *Zmbt1-1::MitTPr-*Δ*TP-ZmBT1-1* and *Zmbt1-1* seeds. The number of proteins that are up- and down-regulated in *Zmbt1-1::MitTPr-*Δ*TP-ZmBT1-1* endosperms is indicated by gray and black bars, respectively. Proteins are indicated by their accession numbers. Their descriptions are shown in Supplemental Table 3. Proteins discussed here are boxed. Proteins that are differentially expressed in WT and *Zmbt1-1* seeds are highlighted in red.

The 118 DEPs with known functions that were differentially expressed in *Zmbt1-1* and *Zmbt1-1::MitTPr-*Δ*TP-ZmBT1-1* endosperms were assembled into 17 functional groups (Figure 5). Notably, mitochondrial delivery of ZmBT1-1 in *Zmbt1-1* strongly up-regulated the expression of SH1 and xylose isomerase, and down-regulated the expresions of CWI-2, SDH, FK, glycolytic, and TCA cycle enzymes (Supplemental Table 3, Figure 5). Also, mitochondrial-specific delivery of ZmBT1-1 in *Zmbt1-1* up-regulated the expression of numerous starch metabolism enzymes [e.g., granule-bound starch synthase, several SSS isoforms, starch phosphorylase, and the small and large subunits of AGP (BT2a and SH2, respectively)] (Supplemental Table 3, Figure 5).

### Lack of Mitochondrial ZmBT1-1 Impedes Down-Regulation of CWI-2 Expression and Up-Regulation of SH1 Expression During Endosperm Development

**Figure 6:**
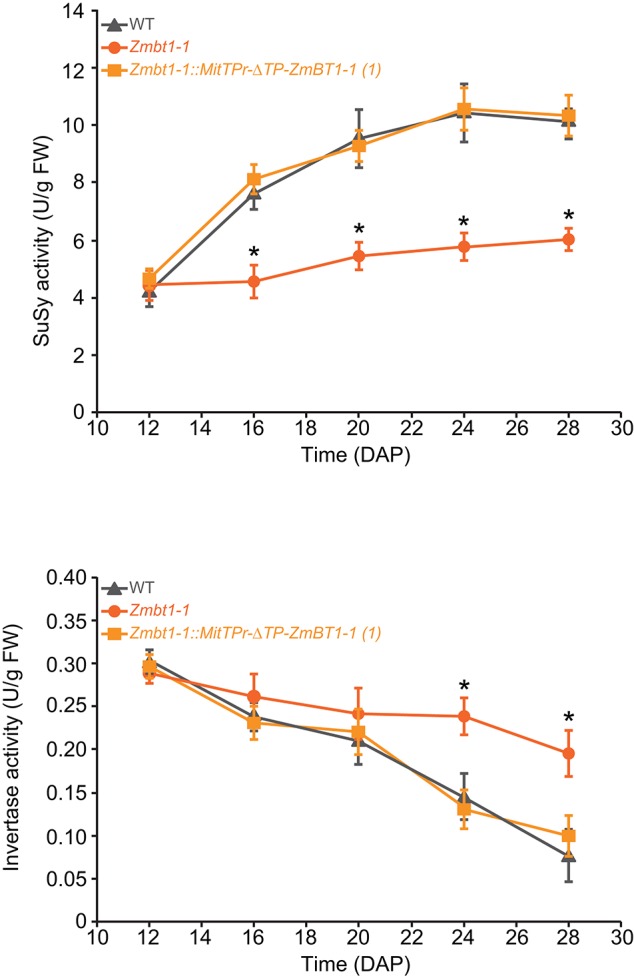
Lack of mitochondrial ZmBT1-1 impedes down-regulation of CWI-2 expression and up-regulation of SH1 expression during endosperm development. The graphics represent total invertase and SuSy activities in WT, *Zmbt1-1*, and *Zmbt1-1::MitTPr-*Δ*TP-ZmBT1-1(1)* endosperms during seed development. Values represent the mean ± SD of determinations on four independent samples. Asterisks indicate significant differences based on Student's *t*-tests. (*P* < 0.05, *Zmbt1-1* mutants vs. WT).

In maize, the level of expression of CWI-2 is greatest early during seed development and it then drops from the 12 DAP stage (Cheng et al., 1996; Prioul et al., 2008) whereas SH1 expression increases from the 14 DAP developmental stage (Doehlert et al., 1988; Méchin et al., 2007; Prioul et al., 2008; Li et al., 2013). The results of the differential proteomic analyses described above suggested that a lack of mitochondrial ZmBT1-1 could result in impairment of the transition from CWI-2- to SH1- mediated sucrose breakdown during endosperm development. To test this hypothesis we measured total invertase and SuSy activities during the development of WT, *Zmbt1-1* and *Zmbt1-1::MitTPr-*Δ*TP-ZmBT1-1* endosperms. As shown in Figure 6 these analyses revealed that, unlike WT and *Zmbt1-1::MitTPr-*Δ*TP-ZmBT1-1* endosperms, total invertase and SuSy activities did not vary much during development of *Zmbt1-1* endosperms.

### Lack of Mitochondrial *ZmBT1-1* Promotes the Accumulation of High Levels of Ethanol and Alanine

**Figure 7:**
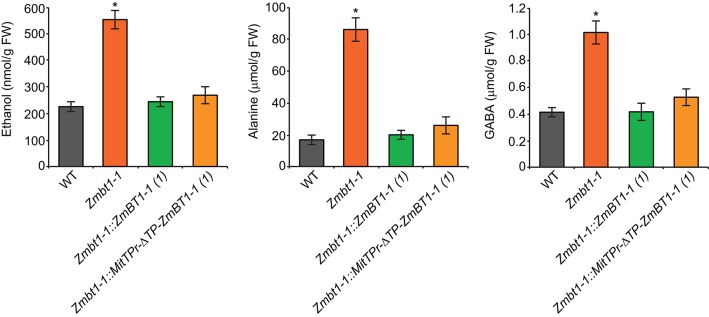
Lack of mitochondrial ZmBT1-1 promotes the accumulation of high levels of ethanol, GABA, and alanine. The graphics represent the contents of the metabolites indicated in 24 DAP endosperms from WT, *Zmbt1-1, Zmbt1-1::ZmBT1-1(1)*, and *Zmbt1-1::MitTPr-*Δ*TP-ZmBT1-1(1)* seeds. Values represent the mean ± SD of determinations on four independent samples. Asterisks indicate significant differences based on Student's *t*-tests. (*P* < 0.05, *Zmbt1-1* mutants vs. WT).

The proteomic analyses suggested that a lack of ZmBT1-1 could promote the accumulation of ethanol and alanine from glycolytically produced pyruvate, as expression of glycolytic and ethanolic fermentation enzymes, and TCA cycle enzymes involved in GABA shunt-mediated alanine production, were higher in *Zmbt1-1* endosperms than in WT endosperms. This inference was corroborated by the analysis of the ethanol, GABA and alanine contents of 24 DAP WT, *Zmbt1-1*, and *Zmbt1-1::ZmBT1-1* endosperms. As shown in Figure 7, the levels of these compounds were higher in *Zmbt1-1* endosperms than in WT and *Zmbt1-1::ZmBT1-1* endosperms. To test the possibility that the absence of mitochondrial ZmBT1-1 causes ethanol, GABA, and alanine over-accumulation in *Zmbt1-1* endosperms we characterized *Zmbt1-1::MitTPr-*Δ*TP-ZmBT1-1* endosperms. We observed that delivering ZmBT1-1 specifically to mitochondria rescues the WT levels of ethanol, GABA, and alanine in *Zmbt1-1* endosperms (Figure 7).

### Lack of Mitochondrial ZmBT1-1 Is Associated With Aberrant Ultrastructural Development in the Mitochondria of Maize Endosperms

Down-regulation of storage metabolism, up-regulation of glycolytic and ethanolic fermentation, and alanine accumulation promoted by the lack of mitochondrial ZmBT1 resemble the responses of plants to conditions in which mitochondrial functioning is compromised (Miyashita and Good, 2008; Shingaki-Wells et al., 2014). Under such conditions, mitochondria fail to develop normally and exhibit signs of degeneration such as loss of cristae, clarification of the matrix and swelling (Shingaki-Wells et al., 2014). To investigate whether the metabolic disorder observed in *Zmbt1-1* endosperms was associated with aberrant mitochondrial development, we analyzed the ultrastructure of mitochondria in 24 DAP WT and *Zmbt1-1* endosperms using ultrafast high-pressure freezing fixantion and transmission electron microscopy. As a control, we also analyzed leaf mitochondria.

**Figure 8:**
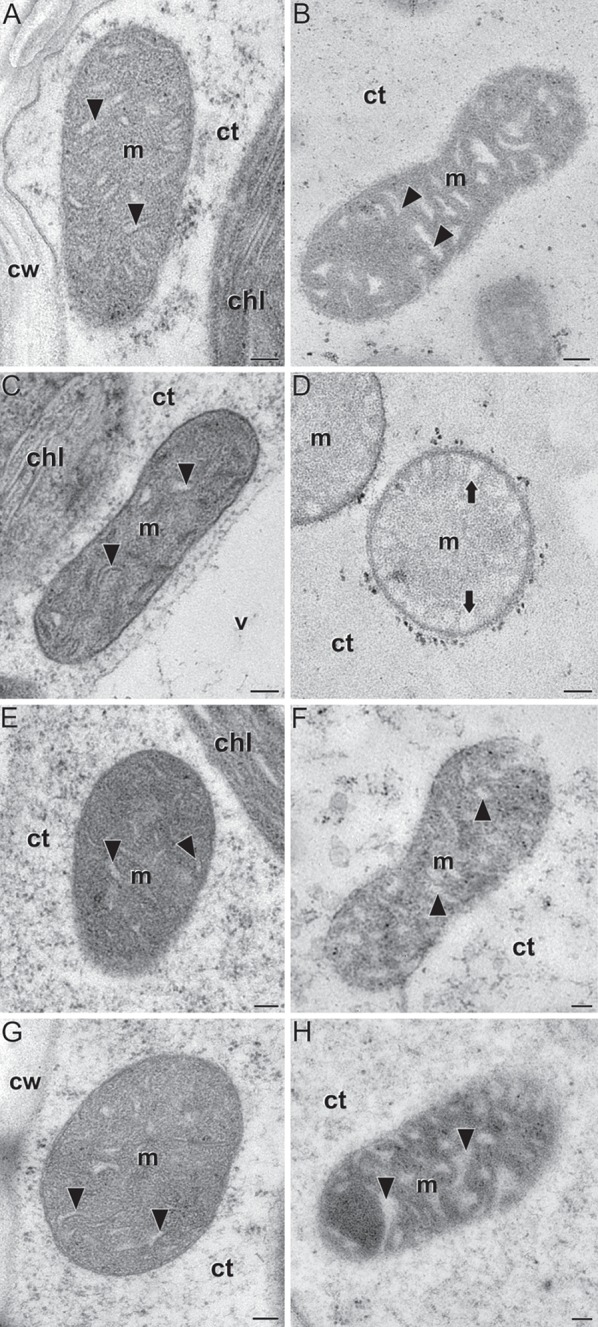
Ultrastructure of mitochondria (m) in leaves **(A,C,E,G)** and 24 DAP endosperms **(B,D,F,H)** of WT **(A,B)**, *Zmbt1-1* **(C,D)**, *Sh2* **(E,F)**, and *Zmbt1-1::MitTPr-*Δ*TP-ZmBT1-1(1)* seeds **(G,H)**. Arrowheads point to mitochondrial cristae. Note their absence in **(D)**, where only round, peripheral invaginations (arrows) are observed. chl, chloroplast; ct, cytoplasm; cw, cell wall; v, vacuole. Bars: 100 nm.

As shown in Figures 8A,C mitochondria of leaves from the two genotypes exhibited a similar, conventional morphology, being oval or elongated, and having conspicuous cristae distributed throughout the matrix. In striking contrast, whereas mitochondria of WT endosperms were elongated and had cristae (Figure 8B, Supplemental Figure 5A), the vast majority of mitochondria in *Zmbt1-1* endosperms were round-oval in shape and had no cristae in the matrix (Figure 8D, Supplemental Figure 5B). In *Zmbt1-1* endosperm mitochondria, only small, round cristae-like invaginations were identified at the organelle periphery closely apposed to the inner membrane (Figure 8D). To investigate whether the ultrastructural differences in mitochondria of *Zmbt1-1* endosperms could be a consequence of reduced starch content, we conducted high-pressure freezing and transmission electron microscopy analyses of 24 DAP endosperms of *Sh2*, a starch-deficient mutant lacking the large SH2 subunit of AGP (Bhave et al., 1990).

As shown in Figures 8E,F mitochondria of *Sh2* leaves and endosperms exhibited conventional morphology, indicating that the aberrant morphology of *Zmbt1-1* endosperm mitochondria is not due to reduced starch.

We next addressed the possibility that the aberrant ultrastructure of mitochondria in *Zmbt1-1* endosperms might be related to the absence of mitochondrial ZmBT1-1. To this end we analyzed mitochondria from *Zmbt1-1::MitTPr-*Δ*TP-ZmBT1-1* plants. As shown in Figures 8G,H *Zmbt1-1::MitTPr-*Δ*TP-ZmBT1-1* leaf and endosperm mitochondria exhibited a ultrastructure indistinguishable from that of WT leaf and endosperm mitochondria. Overall, the data showed that a lack of mitochondrial ZmBT1-1 expression results in aberrant development and architecture of the mitochondria in maize endosperm.

## Discussion

### *ZmBT1-1* Is an Important Determinant of the Metabolic Fate of Incoming Sucrose in Developing Maize Endosperms

The channeling of incoming sucrose into sink metabolism in the maize endosperm requires its cleavage by invertase and SuSy. In maize, the major endosperm invertase, CWI-2, is involved in the transport of photoassimilates into the developing kernel, and its expression is highest early in seed development in the lower endosperm (Cheng et al., 1996; Prioul et al., 2008). The main SuSy isoform in maize endosperms, SH1, is involved in cellulose and starch synthesis (Shannon et al., 1996; Chourey et al., 1998; Thévenot et al., 2005). During seed development, SH1 is expressed first in the upper endosperm; expression then extends to the lower endosperm (Doehlert et al., 1988; Prioul et al., 2008). Like that of ZmBT1-1, SH1 expression increases from the 14 DAP developmental stage and parallels starch accumulation (Doehlert et al., 1988; Méchin et al., 2007; Prioul et al., 2008; Li et al., 2013). Increased SuSy activity during endosperm development can therefore be regarded as a marker for the onset of endosperm starch filling (Prioul et al., 2008).

**Figure 9:**
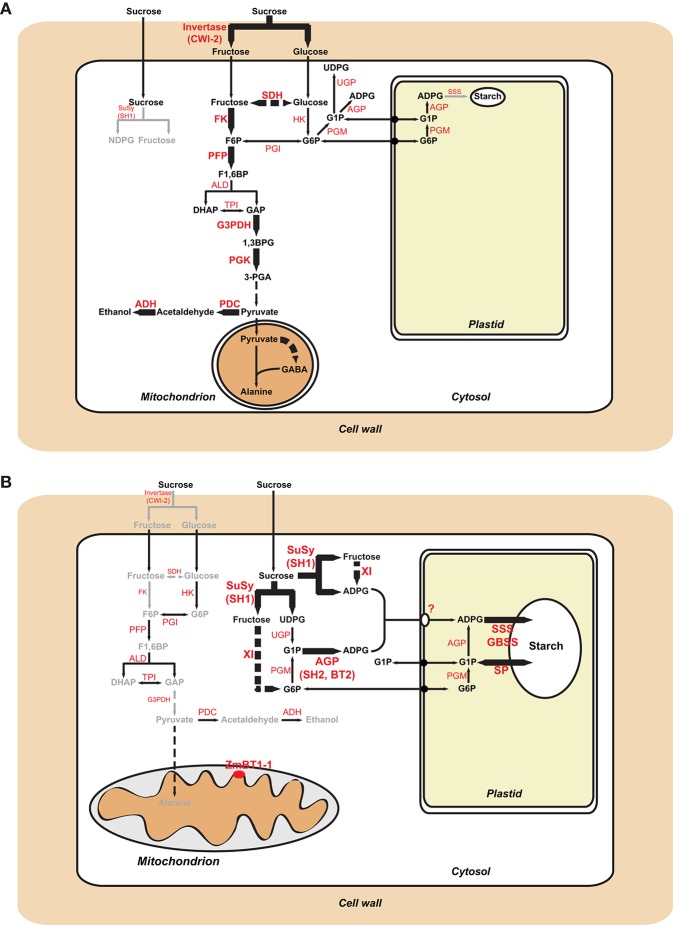
Metabolic schemes illustrating sucrose metabolism pathways in maize endosperms. Panel **(A)** illustrates differences between *Zmbt1-1* and WT endosperms. Enzymatic activities and pathways that are up-regulated in *Zmbt1-1* endosperms are highlighted with large letters and thick arrows, respectively, whereas enzymatic activities and pathways that are down-regulated in *Zmbt1-1* endosperms are highlighted with small letters and gray arrows, respectively. Panel **(B)** illustrates differences between *Zmbt1-1* and *Zmbt1-1::MitTPr-*Δ*TP-ZmBT1-1* endosperms. Enzymatic activities and pathways that are up-regulated in *Zmbt1-1::MitTPr-*Δ*TP-ZmBT1-1* endosperms are highlighted with large, black arrows, whereas enzymatic activities and pathways that are down-regulated in *Zmbt1-1::MitTPr-*Δ*TP-ZmBT1-1* endosperms are highlighted with small letters and gray arrows, respectively. In **(B)**, note the operation of an as-yet unidentified plastidial transporter (?) that facilitates the incorporation of cytosolic ADPglucose into the amyloplast of *Zmbt1-1::MitTPr-*Δ*TP-ZmBT1-1* endosperms. ADH, alcohol dehydrogenase; ALD, aldolase; G3PDH, glyceraldehyde-3-phosphate dehydrogenase; GBSS, granule-bound starch synthase; HK, hexokinase; PDC, pyruvate decarboxylase; PFP, pyrophosphate–fructose-6-phosphate 1-phosphotransferase; PGI, glucose-6-phosphate isomerase; PGK, phosphoglycerate kinase; PGM, phosphoglucomutase; SP, starch phosphorylase; TPI, triose-phosphate-isomerase; UGP, UDPglucose pyrophosphorylase; XI, xylose isomerase.

Differential proteomic analysis of developing *Zmbt1-1* and WT endosperms indicated that glycolytic metabolism of sucrose breakdown products generated by CWI-2, and their subsequent conversion into ethanol and alanine, is more active in *Zmbt1-1* than in WT endosperms, as expression levels of CWI-2, SDH, FK, and enzymes of glycolysis, ethanolic fermentation and the TCA cycle were higher in *Zmbt1-1* endosperms than in WT endosperms (Supplemental Table 1, Figure 4). Furthermore, expression level of alanine aminotransferase was lower in *Zmbt1-1* endosperms than in WT endosperms (Supplemental Table 1, Figure 4). These analyses also indicated that channeling of sucrose into starch metabolism is less active in *Zmbt1-1* than in WT endosperms, as expression levels of SH1 and several SSS isoforms in *Zmbt1-1* endosperms were lower than in WT endosperms (Supplemental Table 1, Figure 4). It thus appears that ZmBT1-1 expression is an important determinant of the switch from invertase- to SuSy-mediated metabolism of the incoming sucrose in maize endosperms. This idea, which is schematically illustrated in Figure 9A, is supported by the fact that the contents of ethanol, alanine and its precursor GABA in developing *Zmbt1-1* endosperms are higher than those in WT endosperms (Figure 7). The hypothesis is given further weight by the results of analyzing total invertase and SuSy activities during the development of WT and *Zmbt1-1* endosperms, which show that, in contrast to WT endosperms, total invertase and SuSy activities do not vary much during the development of *Zmbt1-1* endosperms (Figure 6). The fact that starch accumulates only in the upper part of developing *Zmbt1-1* endosperms (Figure 2D) would strongly indicate that the reduced starch content in this mutant is partly due to impairments in the developmental activation of SH1 expression in the lower part of the endosperm.

### Mitochondrial *ZmBT1-1* Is a Deciding Factor in Endosperm Primary Metabolism

The results presented in Figure 2, which show that *Zmbt1-1::*Δ*TP-ZmBT1-1* and *Zmbt1-1::MitTPr-*Δ*TP-ZmBT1-1* endosperms accumulate WT levels of starch, demonstrate that the delivery of ZmBT1-1 specifically to mitochondria is enough to complement the “low starch content” phenotype of *Zmbt1-1* endosperms. Differential proteomic analysis of developing *Zmbt1-1* and *Zmbt1-1::MitTPr-*Δ*TP-ZmBT1-1* endosperms showed that mitochondrial expression of ZmBT1-1 in *Zmbt1-1* endosperms up-regulates the expression of enzymes involved in the sucrose-to-starch conversion process (i.e., SH1, AGP, starch phosphorylase, SSS, and granule-bound starch synthase), and down-regulates the expression of CWI-2, SDH, FK, and glycolytic enzymes (Supplemental Table 3, Figure 5). These data indicate that, as schematically illustrated in Figure 9B, delivery of ZmBT1-1 specifically to mitochondria in *Zmbt1* endosperms reduces the glycolytic conversion of sucrose breakdown products generated by CWI-2 into ethanol and alanine, and enhances the SH1-mediated sucrose-to-starch conversion pathway. It therefore appears that mitochondrial ZmBT1-1 plays a key role in the transition from invertase- to SuSy-mediated metabolism of the incoming sucrose during endosperm development and thus acts as a major determinant of the metabolic fate of incoming sucrose. In support of this view, analyses of enzymatic activities during endosperm development revealed that the delivery of ZmBT1-1 specifically to mitochondria restored the WT patterns of total invertase and SuSy activities (Figure 6). Furthermore, metabolic analyses showed that levels of ethanol, GABA, and alanine in 24 DAP *Zmbt1-1::MitTPr-*Δ*TP-ZmBT1-1* endosperms were lower than those in *Zmbt1-1* endosperms, and similar to those of WT endosperms (Figure 7). Notably, these analyses also revealed that mitochondrial expression of ZmBT1-1 rescued WT contents of starch, ADPglucose, and other metabolic intermediates of the sucrose-to-starch conversion process in *Zmbt1-1* endosperms (Figures 2, 3).

### Possible Involvement of an As-yet Unidentified Plastidial ADPglucose Transporter in the Sucrose-To-Starch Conversion Process in *Zmbt1-1* Endosperms Expressing ZmBT1-1 Delivered Specifically to Mitochondria

*Zmbt1-1* endosperms accumulate high levels of ADPglucose in the cytosol, which can be due to reduced ZmBT1-1-mediated transport of ADPglucose from the cytosol to the amyloplast (Shannon et al., 1996). Plastidial ZmBT1-1-lacking *Zmbt1-1::*Δ*TP-ZmBT1-1* and *Zmbt1-1::MitTPr-*Δ*TP-ZmBT1-1* endosperms accumulate WT levels of ADPG and starch (Figure 3), which indicates that plastidial ZmBT1-1 is not strictly required for normal starch production in maize endosperms. One explanation to this phenomenon could be that specific delivery of ZmBT1-1 to mitochondria promotes the transit of cytosolic G6P into the amyloplast for its subsequent conversion to starch as schematically illustrated in Figure 9B. In such case, however, high ADPG levels would be expected to occur in *Zmbt1-1::*Δ*TP-ZmBT1-1* and *Zmbt1-1::MitTPr-*Δ*TP-ZmBT1-1* endosperm cells. Alternatively, the enhancement of starch content and the reduction of ADPG content to WT levels in *Zmbt1-1* endosperms by specific delivery of ZmBT1-1 to mitochondria could be due to the fact that delivery of ZmBT1-1 to the mitochondrial compartment of *Zmbt1-1* endosperm cells causes the up-regulation of the expression (or the activation) of an as-yet unidentified plastidial ADPglucose transporter enabling the transport of ADPglucose from the cytosol to the amyloplast. At present it is not possible to draw any firm conclusions as to the class of the translocator responsible for moving ADPglucose across the amyloplast envelope membrane of *Zmbt1-1::*Δ*TP-ZmBT1-1* and *Zmbt1-1::MitTPr-*Δ*TP-ZmBT1-1* endosperms. However, we must emphasize that amyloplasts from plants and organs other than cereal endosperms are capable of transporting ADPglucose (Pozueta-Romero et al., 1991; Naeem et al., 1997). Furthermore, amyloplasts isolated from *bt1* maize and rice endosperms still possess 25% of the capacity of WT amyloplasts to transport ADPglucose (Shannon et al., 1998; Cakir et al., 2016). This residual activity appears to be catalyzed by a uniporter system, as ADPglucose incorporation into isolated amyloplasts of *bt1* rice endosperms is not stimulated by preincubation with ADP (Cakir et al., 2016). Recent studies have shown that nucleoside transporters are capable of transporting extracellular ADPglucose into bacteria (Almagro et al., 2018). Therefore, it is tempting to speculate that plastidial nucleoside transporters could be involved, at least partly, in the transport of ADPglucose across the envelope membrane of *Zmbt1-1::*Δ*TP-ZmBT1-1* and *Zmbt1-1::MitTPr-*Δ*TP-ZmBT1-1* endosperms.

### Mitochondrial *ZmBT1-1* Plays an Important Role in Mitochondrial Function and Its Absence Invokes Retrograde Signaling

Mitochondria are sources of specific signaling molecules that relay information on their energetic and metabolic status to the nucleus. Under conditions in which mitochondrial functioning is compromised, the cell is capable of modulating the expression of nuclear-encoded proteins through a retrograde regulation process, so as to partially counteract the energy crisis that ensues (Rhoads and Subbaiah, 2007; Chandel, 2014). For instance, under conditions of oxygen deprivation, or when the expression of proteins that are important for proper mitochondrial function is compromised, plants down-regulate storage metabolism, up-regulate glycolysis to maintain ATP synthesis, enhance ethanolic fermentative metabolism to regenerate the NAD necessary for glycolysis, and accumulate alanine to store carbon and nitrogen, regulate intracellular pH balance or prevent pyruvate and lactate accumulation (Wiseman et al., 1977; Paumard, 2002; Miyashita and Good, 2008; Busi et al., 2011; Shingaki-Wells et al., 2014). Under such conditions, mitochondria fail to develop normally and exhibit signs of degeneration (Shingaki-Wells et al., 2014). Results presented in this work provide strong evidence that mitochondrial ZmBT1-1 plays an important role in mitochondrial function, energy provision and primary metabolism in maize endosperms, and indicate that its absence invokes changes in the expression of nuclear-encoded proteins to compensate for the associated metabolic perturbations. This is supported by facts showing that (i) vast majority of mitochondria in *Zmbt1-1* endosperms are aberrant (Figure 8), (ii) *Zmbt1-1* endosperms accumulate low levels of starch and high levels of ethanol and alanine in *Zmbt1-1* endosperms (Figures 3, 7) due to altered expression of nuclear-encoded starch biosynthetic enzymes and enzymes involved in glycolysis and ethanol and alanine production (Figure 4, Supplemental Table 1); and (iii) delivering ZmBT1-1 specifically to mitochondria reverts the *Zmbt1-1* phenotype and protein expression pattern (Figures 2–8, Supplemental Table 3).

### A Suggested Functional Role for Mitochondrial ZmBT1-1 in Facilitating Exchange Between Intramitochondrial AMP and Cytosolic ADP in Maize Endosperms

Dual targeting of proteins to mitochondria and plastids occurs mainly when proteins have overlapping functions in the two organelles (Smith et al., 1998; Peeters and Small, 2001; Goggin et al., 2003; Christensen et al., 2005; Duchêne et al., 2005; Kmiec et al., 2014), although dually targeted proteins may also fulfill different roles in plastids and mitochondria. In some cases, proteins with dual targeting play important roles in mitochondria, but not in plastids (Tarasenko et al., 2016).

**Figure 10:**
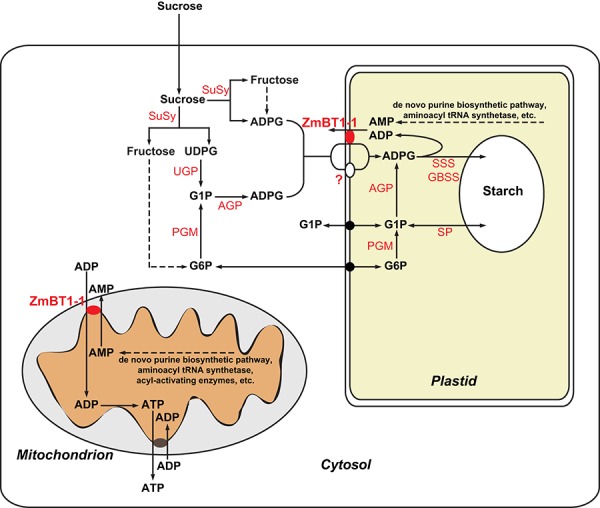
Metabolic scheme illustrating the suggested roles of ZmBT1-1 in the mitochondrion and in the amyloplast of maize endosperms. In amyloplasts, ZmBT1-1 could play a role in facilitating the incorporation of cytosolically produced ADPglucose in exchange for newly synthesized AMP or ADP produced by the starch synthase reaction. In mitochondria, ZmBT1-1 could play a decisive role in exporting AMP to the cytosol in exchange for ADP which, once in the matrix, could be converted to ATP by oxidative phosphorylation. ATP generated in the mitochondrion could be exported to the cytosol by means of the ADP/ATP carrier. GBSS, granule-bound starch synthase; PGM, phosphoglucomutase; SP, starch phosphorylase; UGP, UDP glucose pyrophosphorylase.

ZmBT1-1 and homologs in other cereal species are counter-exchange transporters that recognize not only ADPglucose, but also ADP and AMP (Shannon et al., 1998; Bowsher et al., 2007; Cakir et al., 2016). Accordingly, as illustrated in Figure 10, we propose that in maize endosperms ZmBT1-1 could play a decisive role in exporting AMP from mitochondria to the cytosol in exchange for ADP. Such transporters are not without precedent, since mitochondrial AMP exporters have been reported in both yeast and mammals (Fiermonte et al., 2004; Todisco et al., 2006). Possible sources of AMP in the mitochondrial matrix include reactions leading to the activation of amino acids for protein synthesis, and formation of various CoA-derivatives (catalyzed by amino-acyl tRNA synthetases and acyl-activating enzymes, respectively) (Shockey et al., 2003; Duchêne et al., 2005; Igamberdiev and Kleczkowski, 2006). Although it has been suggested that *de novo* purine biosynthesis that leads to AMP production is exclusively located in plastids (Zrenner et al., 2006), immunolocalization and proteomic analyses have provided evidence that enzymes of the *de novo* purine biosynthesis pathway are also localized to mitochondria in several organs and species (Atkins et al., 1997; Goggin et al., 2003; cf. Supplemental Table 3, Krath and Hove-Jensen, 1999; Huang et al., 2009; cf. Supplemental Table 1, Lee et al., 2013).

ADP incorporated into the matrix by mitochondrial ZmBT1-1 could be converted to ATP to be exported to the cytosol by means of the ADP/ATP carrier (Figure 10). Thus, the net balance inherent in the suggested mechanism of ZmBT1-1-mediated adenylate transport in maize endosperms would imply the export to the cytosol of one molecule each of mitochondrially synthesized AMP and ATP in exchange for two molecules of cytosolic ADP (Figure 10). Mitochondrial ZmBT1-1 could therefore play a role in energy provision by mitochondria in maize endosperms. In amyloplasts, ZmBT1-1 could participate in facilitating the incorporation of cytosolic ADPglucose in exchange for newly synthesized AMP or ADP produced by the starch synthase reaction (Figure 10).

## Author Contributions

AB, FM, JS-S, MO, EB-F, and JP-R designed the experiments and analyzed the data. AB, FM, CC-F, AR-S, VP-V, MO, JL, ÁS-L, GA, and EB-F performed most of experiments. AB, JS-S, and JP-R supervised the experiments. AB, JS-S, and JP-R wrote the article.

### Conflict of Interest Statement

The authors declare that the research was conducted in the absence of any commercial or financial relationships that could be construed as a potential conflict of interest.

## Abbreviations

AGP: ADPglucose pyrophosphorylase
CWI-2: Cell wall invertase 2
DAP: days after pollination
DEPs: differentially expressed proteins
FK: fructokinase
G6P: glucose-6-phosphate
MCF: Mitochondrial carrier family
SuSy: sucrose synthase
SSS: soluble starch synthase
SDH: sorbitol dehydrogenase
TP: transit peptide
ZmBT1-1: *Zea mays* Brittle1-1.
