# Supplementary material for: Mitochondrial Zea mays Brittle1-1 Is a Major Determinant of the Metabolic Fate of Incoming Sucrose and Mitochondrial Function in Developing Maize Endosperms
Source: Front Plant Sci. 2019 Mar 12;10:242. doi: 10.3389/fpls.2019.00242 (PMC6423154; doi:10.3389/fpls.2019.00242)
Supplement: Supplementary file 8 [file Presentation_1.pdf]

SUPPLEMENTAL DATA

Supplemental Figure 1: Schematic illustration of the canonical pathway of starch biosynthesis in cereal endosperm cells (Bahaji et al., 2014; Boehlein et al., 2018; Kleczkowski, 1996). According to this interpretation incoming sucrose is cytosolically converted into ADPglucose by the stepwise reactions of SuSy, UDPglucose pyrophosphorylase (UGP) and AGP. ADPglucose can also be directly synthesized by SuSy. ADPglucose incorporated into the amyloplast by means of ZmBT1-1 in exchange for ADP is utilized by SSS and GBSS to produce starch.

Supplemental Figure 2: PCR analyses of WT, homozygous *Zmbt1-1* plants and homozygous *Zmbt1-1* plants transformed with *UBI-ZmBT1-1, UBI-ΔTP-ZmBT1-1* or *UBI-MitTPr-ΔTP-ZmBT1-1* (*Zmbt1-1::ZmBT1-1, Zmbt1-1::ΔTP-ZmBT1-1* and *Zmbt1-1::MitTPr-ΔTP-ZmBT1-1,* respectively). **(A)** Site of *dSpm* insertion in the *Zmbt1-1* knockout line (Maize Genetics COOP Stock Center. Ref. *bt1-m1::dSpm,* 514N). **(B)** PCR analysis of genomic DNA from WT, homozygous *Zmbt1-1, Zmbt1-1::ZmBT1-1, Zmbt1-1::ΔTP-ZmBT1-1* and *Zmbt1-1::MitTPr-ΔTP-ZmBT1-1* plants using the O1 and O2 genomic *ZmBT1-1*-specific primers, and the *dSpm*-specific O3 primer. **(C)** Schematic representation of the *UBI-ZmBT1-1, UBI-ΔTP-ZmBT1-1* and *UBI-MitTPr-ΔTP-ZmBT1-1* constructs used to produce *Zmbt1-1::ZmBT1-1, Zmbt1-1::ΔTP-ZmBT1-1* and *Zmbt1-1::MitTPr-ΔTP-ZmBT1-1,* respectively. **(D)** PCR analyses of genomic DNA from WT, homozygous *Zmbt1-1* plants, *Zmbt1-1::ZmBT1-1, Zmbt1-1::ΔTP-ZmBT1-1* and *Zmbt1-1::MitTPr-ΔTP-ZmBT1-1* plants using the *Ubi-1* promoter-specific O4 primer and the *ZmBT1-1*-specific O5 primer. (E) PCR analyses of genomic DNA from WT, homozygous *Zmbt1-1, Zmbt1-1::ZmBT1-1, Zmbt1-1::ΔTP-ZmBT1-1* and *Zmbt1-1::MitTPr-ΔTP-ZmBT1-1* plants using the *Ubi-1* promoter-specific O4 primer and the O6 primer specific for the ZmBT1-1 plastidial TP encoding sequence. **(F)** PCR analyses of genomic DNA from WT, homozygous *Zmbt1-1, Zmbt1-1::ZmBT1-1, Zmbt1-1::ΔTP-ZmBT1-1* and *Zmbt1-1::MitTPr-ΔTP-ZmBT1-1* plants using the *ZmBT1-1*-specific O5 primer and the O7 primer specific for the MitTPr encoding sequence. O1-6 specific positions are indicated in **(A)** and **(C)**.

Using the O1 and O2 *ZmBT1-1* specific primers, we amplified a ca. 500 bp PCR product from DNA isolated from WT plants (**panel B**). No such fragment could be PCR-amplified from genomic DNA isolated from homozygous *Zmbt1-1* and homozygous *Zmbt1-1::ZmBT1-1, Zmbt1-1::ΔTP-ZmBT1-1* and *Zmbt1-1::MitTPr-ΔTP-ZmBT1-1* plants (**panel B**), indicating (a) the absence of WT genomic *ZmBT1-1,* and (b) the possible occurrence in these plants of a long DNA insertion (*dSpm*) between *ZmBT1-1* genomic sequences that hybridize with O1 and O2. Using O1 and O3, we amplified a 777 bp PCR fragment from DNA of homozygous *Zmbt1-1, Zmbt1-1::ZmBT1-1, Zmbt1-1::ΔTP-ZmBT1-1* and *Zmbt1-1::MitTPr-ΔTP-ZmBT1-1* plants (**panel B**), which confirmed the occurrence of *dSpm* in *ZmBT1-1.* Using O4 and O5 we PCR-amplified 590, 521 and 755 bp PCR products from DNA of *Zmbt1-1::ZmBT1-1, Zmbt1-1::ΔTP-ZmBT1-1* and *Zmbt1-1::MitTPr-ΔTP-ZmBT1-1* plants, respectively (**panel D**). As expected, no such fragments could be amplified from WT and *Zmbt1-1* plants (**panel D**). Moreover, using O4 and O6 we amplified a PCR product of 257 bp from DNA of *Zmbt1-1::ZmBT1-1* plants, but not from WT, *Zmbt1-1, Zmbt1-1::ΔTP-ZmBT1-1* and *Zmbt1-1::MitTPr-ΔTP-ZmBT1-1* plants (**panel E**). Furthermore, using O5 and O7 primers we amplified a PCR product of 569 bp from DNA of *Zmbt1-1::MitTPr-ΔTP-ZmBT1-1* plants, but not from WT, *Zmbt1-1, Zmbt1-1::ZmBT1-1* and *Zmbt1-1:: ΔTP-ZmBT1-1* plants (**panel F**).

Supplemental Figure 3: Stages in the production of constructs used in this work.

Supplemental Figure 4: Delivery of ZmBT1-1 specifically to mitochondria complements the slow germination and delayed growth phenotype of *Zmbt1-1* plants. Photographs illustrate the morphology of the indicated seedlings and plantlets at 7 and 14 days after sowing (A and B, respectively).

Supplemental Figure 5: Ultrastructure of mitochondria (m) in endosperm cells of (A) WT and (B) *Zmbt1-1* 24 DAP seeds. ct, cytoplasm. Bars: 500 nm.

Supplemental Video 1: Movie showing distribution pattern, size, shape and motility of GFP fluorescence in TP-ZmBT1-1-GFP expressing maize leaves.

Supplemental Video 2: Movie showing distribution pattern, size, shape and motility of GFP fluorescence in MitTPr-TP-ZmBT1-1-GFP expressing maize leaves.

Supplemental Video 3: Movie showing distribution pattern, size, shape and motility of GFP fluorescence in MitTPr-GFP expressing maize leaves.

REFERENCES

Bahaji, A., Li, J., Sánchez-López, Á. M., Baroja-Fernández, E., Muñoz, F. J., Ovecka, M., et al. (2014). Starch biosynthesis, its regulation and biotechnological approaches to improve crop yields. *Biotechnol. Adv.* 32, 87–106. doi:10.1016/j.biotechadv.2013.06.006.

Boehlein, S. K., Shaw, J. R., Boehlein, T. J., Boehlein, E. C., and Hannah, L. C. (2018). Fundamental differences in starch synthesis in the maize leaf, embryo, ovary and endosperm. *Plant J.* 96, 595–606. doi:10.1111/tpj.14053.

Kleczkowski, L. A. (1996). Back to the drawing board: redefining starch synthesis in cereals. *Trends Plant Sci.* 1, 363–364.
