# Supplementary material for: Mitochondrial Zea mays Brittle1-1 Is a Major Determinant of the Metabolic Fate of Incoming Sucrose and Mitochondrial Function in Developing Maize Endosperms
Source: Front Plant Sci. 2019 Mar 12;10:242. doi: 10.3389/fpls.2019.00242 (PMC6423154; doi:10.3389/fpls.2019.00242)
Supplement: Supplementary file 9 [file Image_1.pdf]

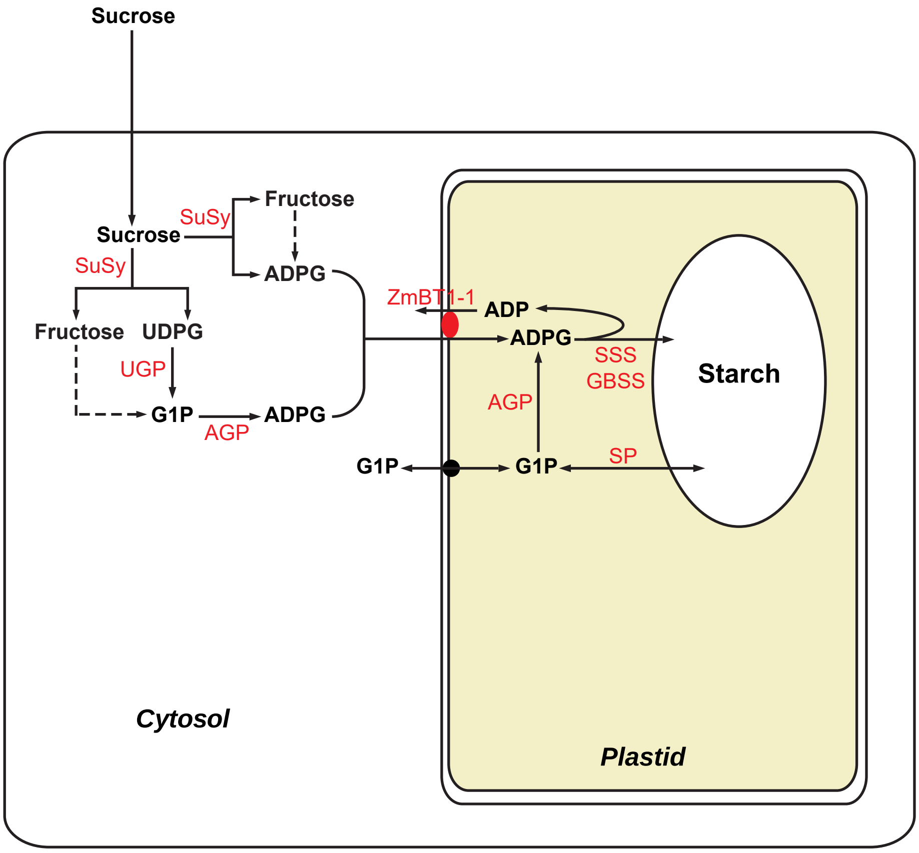

Supplemental Figure 1: Schematic illustration of the canonical pathway of starch biosynthesis in cereal endosperm cells (Bahaji et al., 2014; Boehlein et al., 2018; Kleczkowski, 1996). According to this interpretation incoming sucrose is cytosolically converted into ADPglucose by the stepwise reactions of SuSy, UDPglucose pyrophosphorylase (UGP) and AGP. ADPglucose can also be directly synthesized by SuSy. ADPglucose incorporated into the amyloplast by means of ZmBT1-1 in exchange for ADP is utilized by SSS and GBSS to produce starch.
