# Supplementary material for: Mitochondrial Zea mays Brittle1-1 Is a Major Determinant of the Metabolic Fate of Incoming Sucrose and Mitochondrial Function in Developing Maize Endosperms
Source: Front Plant Sci. 2019 Mar 12;10:242. doi: 10.3389/fpls.2019.00242 (PMC6423154; doi:10.3389/fpls.2019.00242)
Supplement: Supplementary file 10 [file Image_2.pdf]

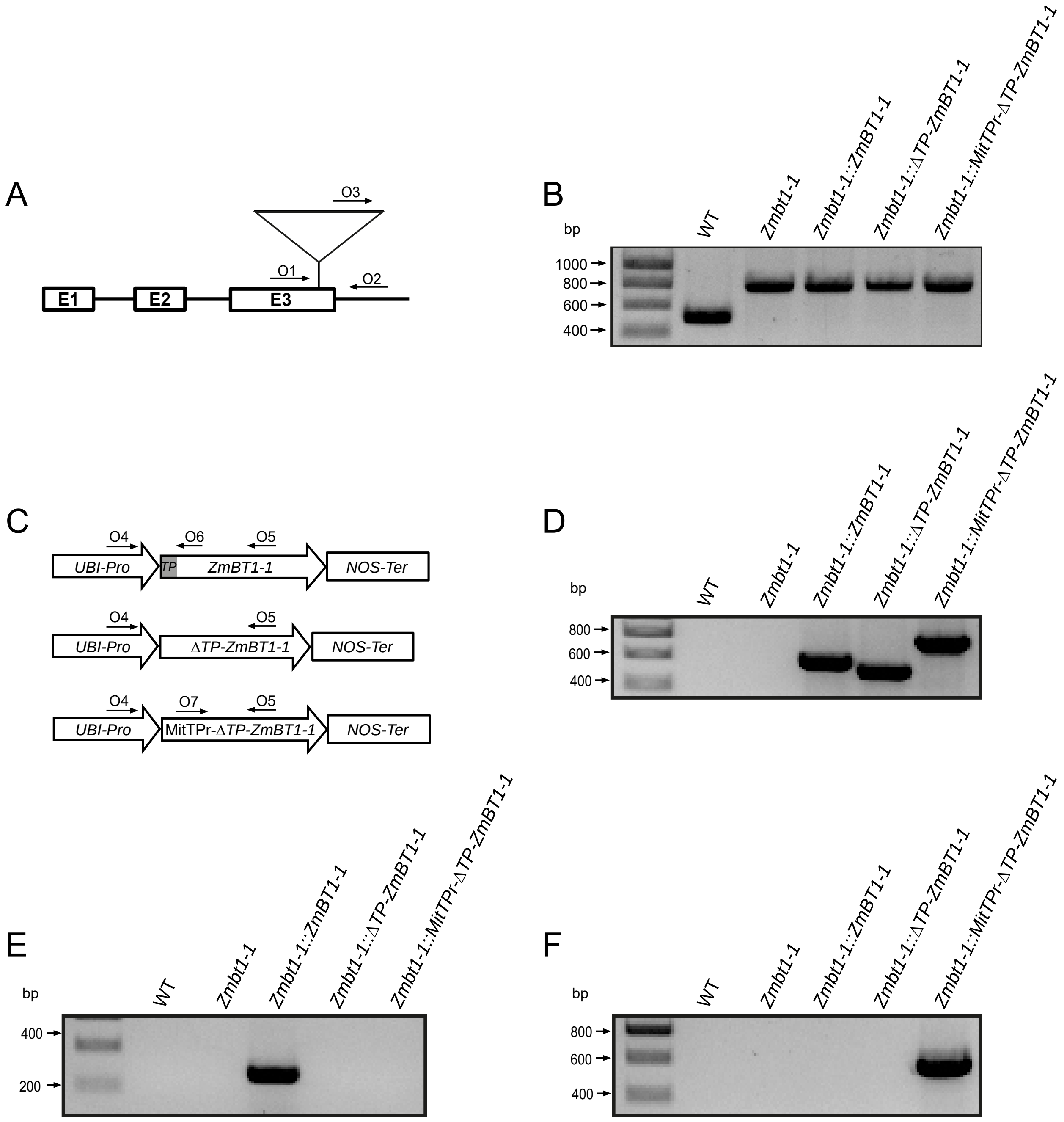

Supplemental Figure 2

Supplemental Figure 2: PCR analyses of WT, homozygous *Zmbt1-1* plants and homozygous *Zmbt1-1* plants transformed with *UBI-ZmBT1-1, UBI-ΔTP-ZmBT1-1* or *UBI-MitTPr-ΔTP-ZmBT1-1* (*Zmbt1-1::ZmBT1-1, Zmbt1-1::ΔTP-ZmBT1-1* and *Zmbt1-1::MitTPr-ΔTP-ZmBT1-1,* respectively). **(A)** Site of *dSpm* insertion in the *Zmbt1-1* knockout line (Maize Genetics COOP Stock Center. Ref. *bt1-m1::dSpm,* 514N). **(B)** PCR analysis of genomic DNA from WT, homozygous *Zmbt1-1*, *Zmbt1-1::ZmBT1-1, Zmbt1-1::ΔTP-ZmBT1-1* and *Zmbt1-1::MitTPr-ΔTP-ZmBT1-1* plants using the O1 and O2 genomic *ZmBT1-1*-specific primers, and the *dSpm*-specific O3 primer. **(C)** Schematic representation of the *UBI-ZmBT1-1*, *UBI-ΔTP-ZmBT1-1* and *UBI-MitTPr-ΔTP-ZmBT1-1* constructs used to produce *Zmbt1-1::ZmBT1-1, Zmbt1-1::ΔTP-ZmBT1-1* and *Zmbt1-1::MitTPr-ΔTP-ZmBT1-1,* respectively. **(D)** PCR analyses of genomic DNA from WT, homozygous *Zmbt1-1* plants, *Zmbt1-1::ZmBT1-1, Zmbt1-1::ΔTP-ZmBT1-1* and *Zmbt1-1::MitTPr-ΔTP-ZmBT1-1* plants using the *Ubi-1* promoter-specific O4 primer and the *ZmBT1-1*-specific O5 primer. **(E)** PCR analyses of genomic DNA from WT, homozygous *Zmbt1-1, Zmbt1-1::ZmBT1-1, Zmbt1-1::ΔTP-ZmBT1-1* and *Zmbt1-1::MitTPr-ΔTP-ZmBT1-1* plants using the *Ubi-1* promoter-specific O4 primer and the O6 primer specific for the ZmBT1-1 plastidial TP encoding sequence. **(F)** PCR analyses of genomic DNA from WT, homozygous *Zmbt1-1, Zmbt1-1::ZmBT1-1, Zmbt1-1::ΔTP-ZmBT1-1* and *Zmbt1-1::MitTPr-ΔTP-ZmBT1-1* plants using the *ZmBT1-1*-specific O5 primer and the O7 primer specific for the MitTPr encoding sequence. O1-6 specific positions are indicated in **(A)** and **(C).**

Using the O1 and O2 *ZmBT1-1* specific primers, we amplified a ca. 500 bp PCR product from DNA isolated from WT plants (**panel B**). No such fragment could be PCR-amplified from genomic DNA isolated from homozygous *Zmbt1-1* and homozygous *Zmbt1-1::ZmBT1-1, Zmbt1-1::ΔTP-ZmBT1-1* and *Zmbt1-1::MitTPr-ΔTP-ZmBT1-1* plants (**panel B**), indicating (a) the absence of WT genomic *ZmBT1-1,* and (b) the possible occurrence in these plants of a long DNA insertion (*dSpm*) between *ZmBT1-1* genomic sequences that hybridize with O1 and O2. Using O1 and O3, we amplified a 777 bp PCR fragment from DNA of homozygous *Zmbt1-1*, *Zmbt1-1::ZmBT1-1, Zmbt1-1::ΔTP-ZmBT1-1* and *Zmbt1-1::MitTPr-ΔTP-ZmBT1-1* plants (**panel B**), which confirmed the occurrence of *dSpm* in *ZmBT1-1.* Using O4 and O5 we PCR-amplified 590, 521 and 755 bp PCR products from DNA of *Zmbt1-1::ZmBT1-1, Zmbt1-1::ΔTP-ZmBT1-1* and *Zmbt1-1::MitTPr-ΔTP-ZmBT1-1* plants, respectively (**panel D**). As expected, no such fragments could be amplified from WT and *Zmbt1-1* plants (**panel D**). Moreover, using O4 and O6 we amplified a PCR product of 257 bp from DNA of *Zmbt1-1::ZmBT1-1* plants, but not from WT, *Zmbt1-1*, *Zmbt1-1::ΔTP-ZmBT1-1* and *Zmbt1-1::MitTPr-ΔTP-ZmBT1-1* plants (**panel E**). Furthermore, using O5 and O7 primers we amplified a PCR product of 569 bp from DNA of *Zmbt1-1::MitTPr-ΔTP-ZmBT1-1* plants, but not from WT, *Zmbt1-1*, *Zmbt1-1::ZmBT1-1* and *Zmbt1-1:: ΔTP-ZmBT1-1* plants (**panel F**).
