# Supplementary material for: Mitochondrial Zea mays Brittle1-1 Is a Major Determinant of the Metabolic Fate of Incoming Sucrose and Mitochondrial Function in Developing Maize Endosperms
Source: Front Plant Sci. 2019 Mar 12;10:242. doi: 10.3389/fpls.2019.00242 (PMC6423154; doi:10.3389/fpls.2019.00242)
Supplement: Supplementary file 11 [file Image_3.pdf]

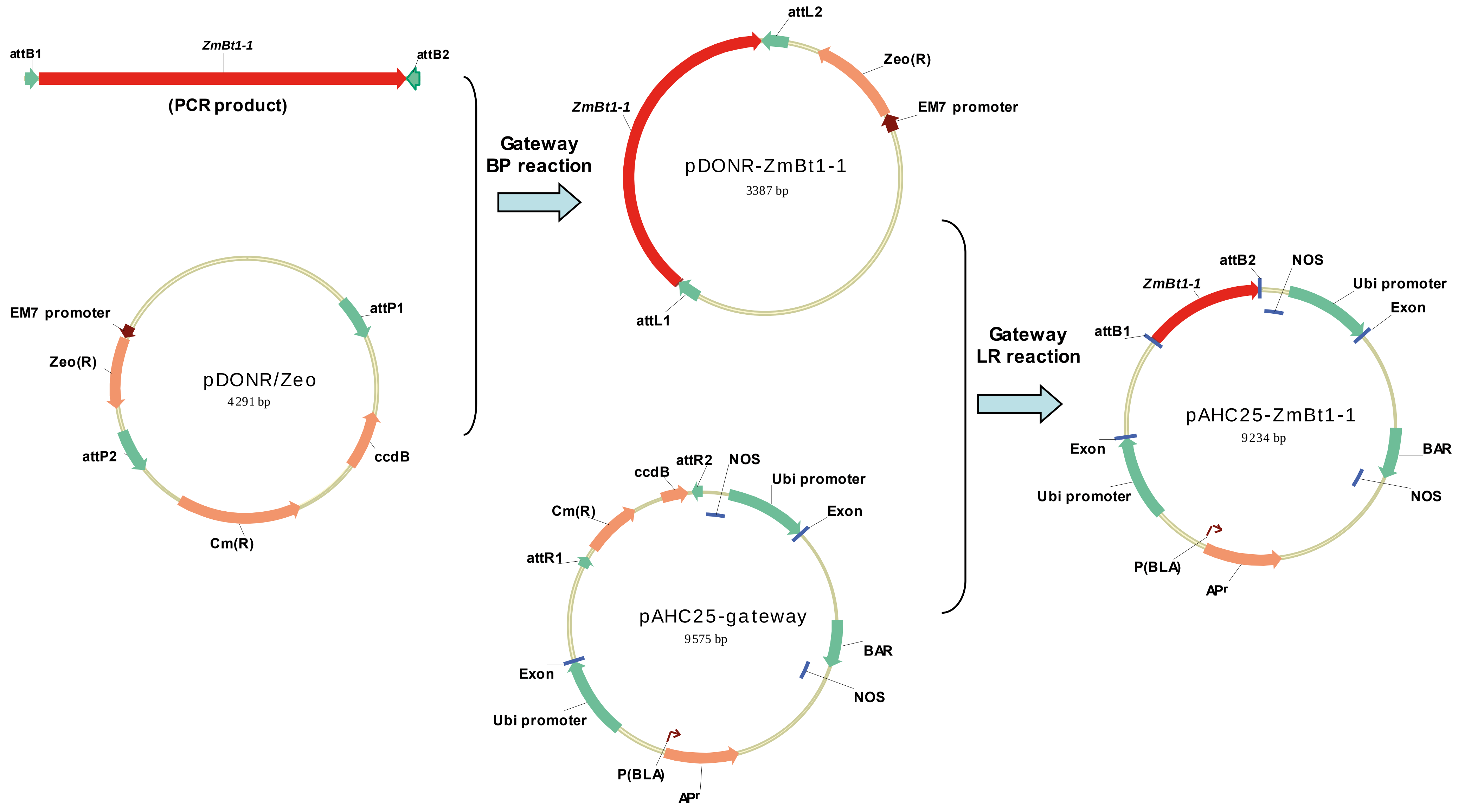

Supplemental Figure 3: Stages in the production of constructs used in this work.

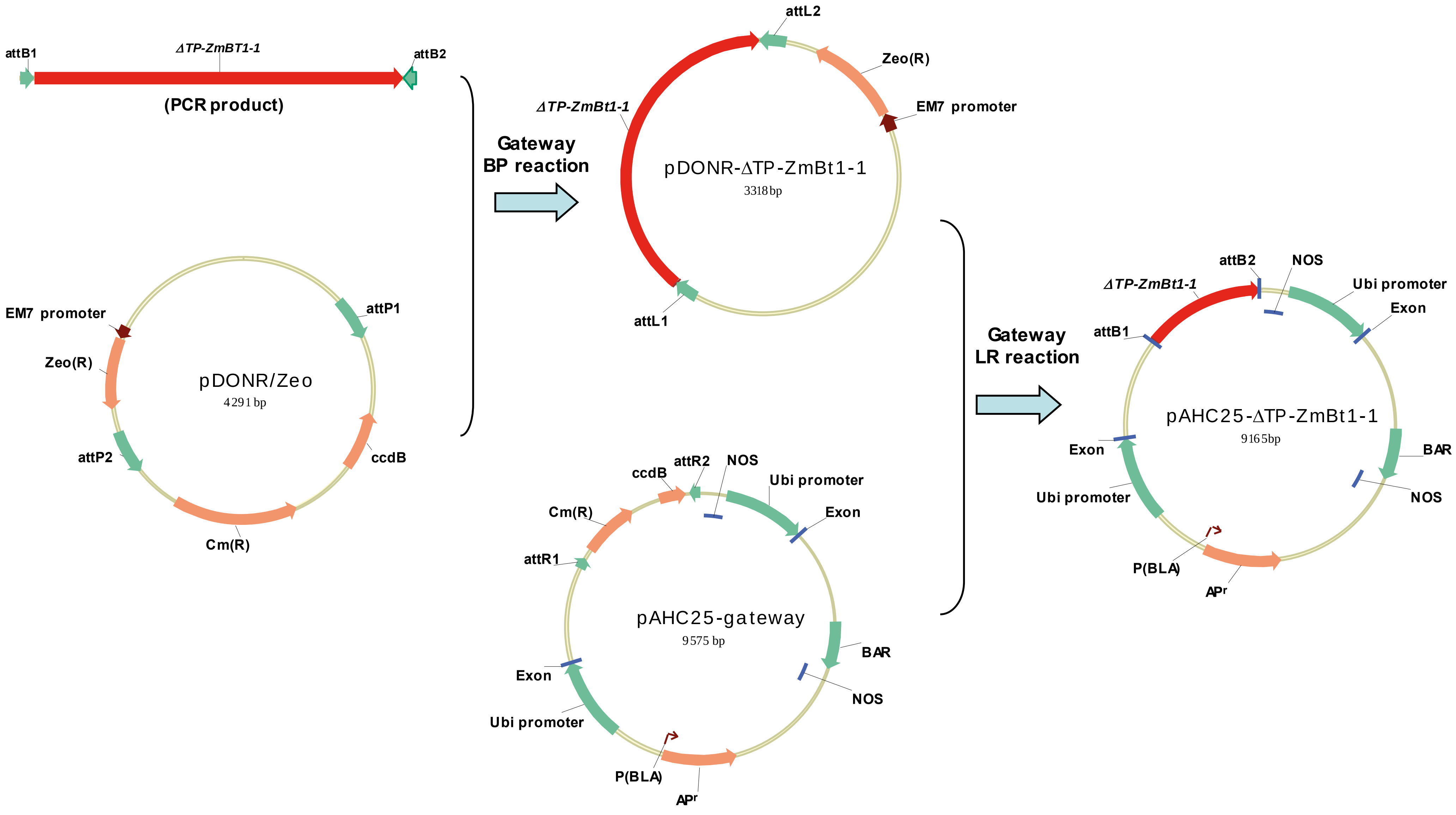
attB1
ΔTP-ZmBT1-1
attB2
(PCR product)
EM7 promoter
attP1
Zeo(R)
pDONR/Zeo
4291 bp
attP2
ccdB
Cm(R)
Gateway
BP reaction
attL2
Zeo(R)
ΔTP-ZmBt1-1
EM7 promoter
pDONR-ΔTP-ZmBt1-1
3318bp
attL1
ccdB
attR2
NOS
Ubi promoter
Cm(R)
Exon
attR1
pAHC25-gateway
9575 bp
BAR
Exon
NOS
Ubi promoter
P(BLA)
AP^r
Gateway
LR reaction
attB2
NOS
ΔTP-ZmBt1-1
Ubi promoter
attB1
Exon
pAHC25-ΔTP-ZmBt1-1
9165bp
Exon
BAR
Ubi promoter
NOS
P(BLA)
AP^r

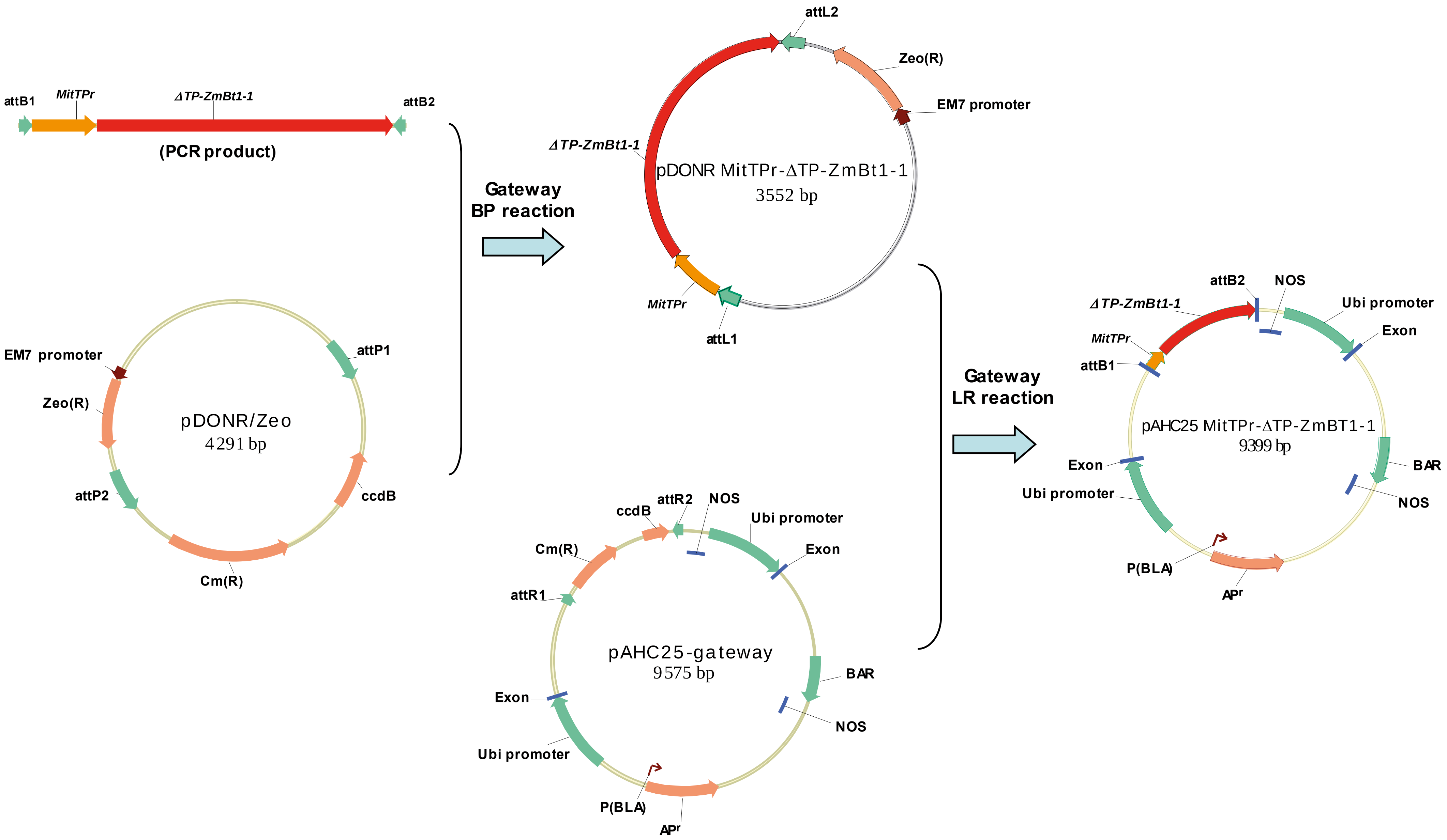
attB1
MitTPr
ΔTP-ZmBt1-1
attB2
(PCR product)
EM7 promoter
attP1
Zeo(R)
pDONR/Zeo
4291 bp
attP2
ccdB
Cm(R)
Gateway
BP reaction
attL2
Zeo(R)
EM7 promoter
ΔTP-ZmBt1-1
pDONR MitTPr-ΔTP-ZmBt1-1
3552 bp
MitTPr
attL1
ccdB
attR2
NOS
Ubi promoter
Cm(R)
Exon
attR1
pAHC25-gateway
9575 bp
BAR
Exon
NOS
Ubi promoter
P(BLA)
AP^r
Gateway
LR reaction
attB2
NOS
ΔTP-ZmBt1-1
Ubi promoter
MitTPr
Exon
attB1
pAHC25 MitTPr-ΔTP-ZmBT1-1
9399 bp
Exon
BAR
Ubi promoter
NOS
P(BLA)
AP^r

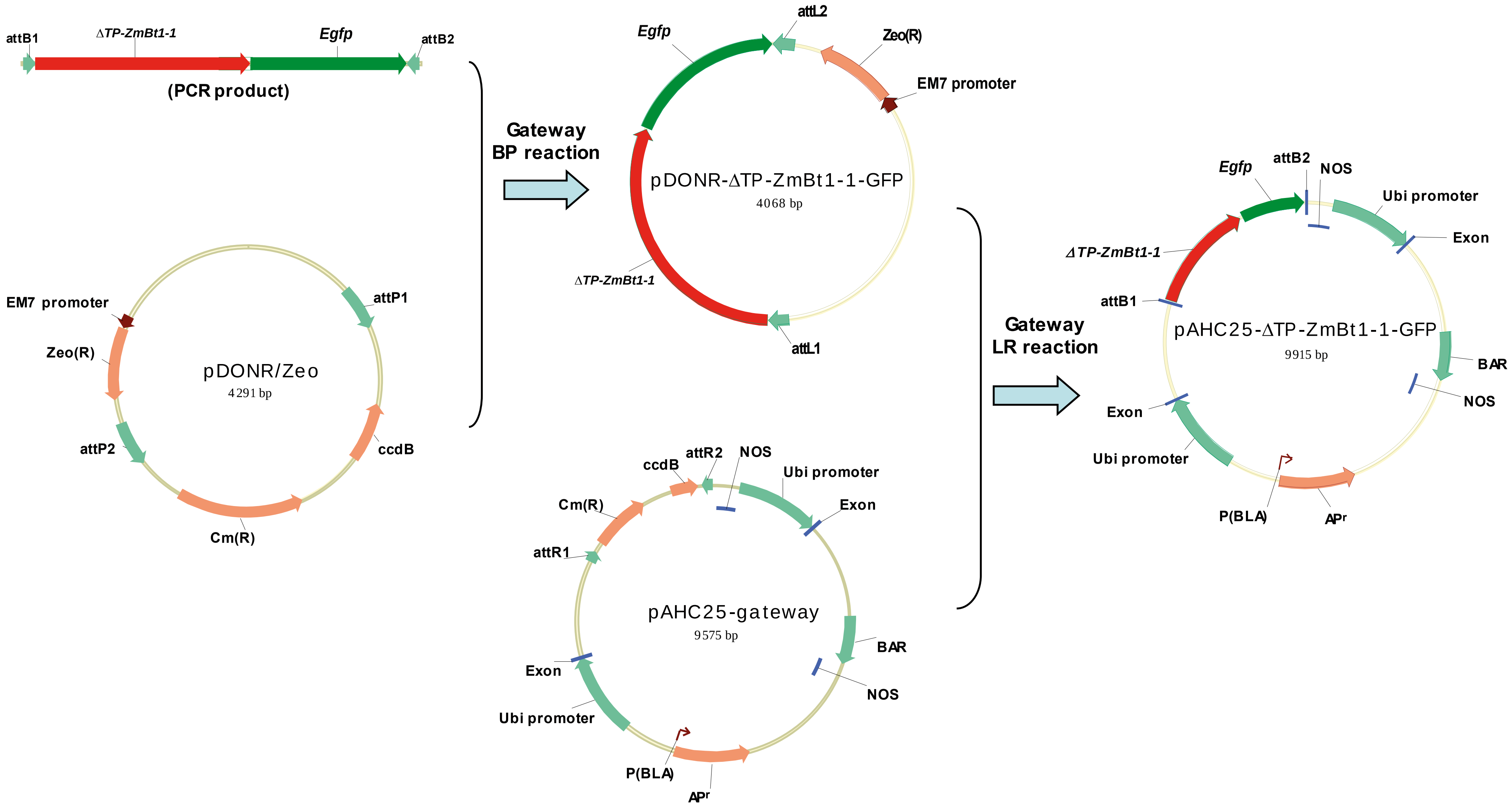

attB1
ΔTP-ZmBt1-1
Egfp
attB2
(PCR product)
EM7 promoter
attP1
Zeo(R)
pDONR/Zeo
4291 bp
attP2
ccdB
Cm(R)
Gateway
BP reaction
Egfp
attL2
Zeo(R)
EM7 promoter
pDONR-ΔTP-ZmBt1-1-GFP
4068 bp
ΔTP-ZmBt1-1
attL1
ccdB
attR2
NOS
Ubi promoter
Cm(R)
Exon
attR1
pAHC25-gateway
9575 bp
Exon
BAR
Ubi promoter
NOS
P(BLA)
APr
Gateway
LR reaction
Egfp
attB2
NOS
Ubi promoter
ΔTP-ZmBt1-1
Exon
attB1
pAHC25-ΔTP-ZmBt1-1-GFP
9915 bp
BAR
Exon
NOS
Ubi promoter
P(BLA)
APr

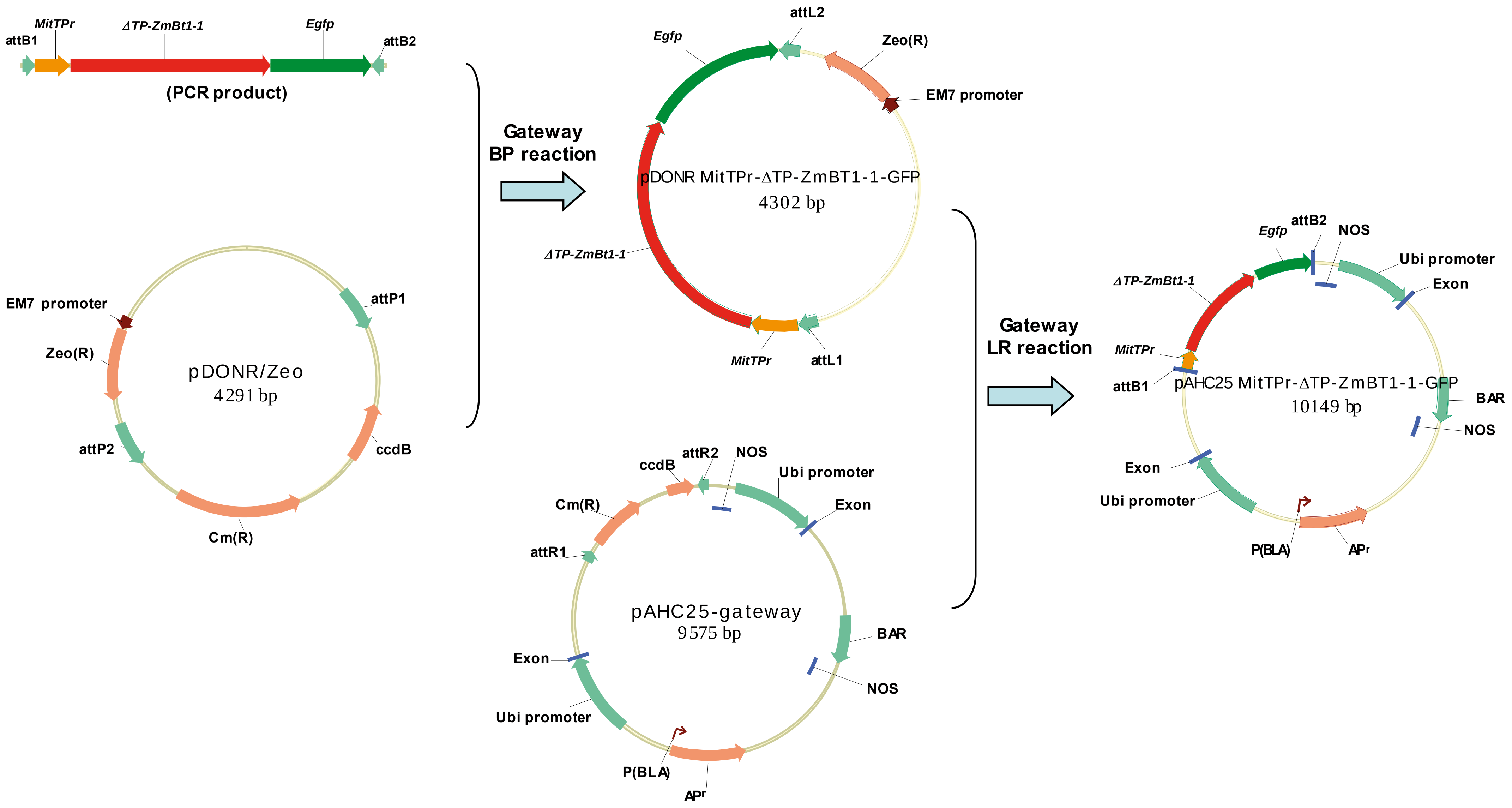
attB1
MitTPr
ΔTP-ZmBt1-1
Egfp
attB2
(PCR product)
EM7 promoter
Zeo(R)
pDONR/Zeo
4291 bp
attP1
attP2
ccdB
Cm(R)
Gateway
BP reaction
pDONR MitTPr-ΔTP-ZmBT1-1-GFP
4302 bp
attL2
Zeo(R)
EM7 promoter
MitTPr
attL1
pAHC25-gateway
9575 bp
ccdB
attR2
NOS
Ubi promoter
Exon
Cm(R)
attR1
BAR
NOS
Exon
Ubi promoter
P(BLA)
APr
Gateway
LR reaction
pAHC25 MitTPr-ΔTP-ZmBT1-1-GFP
10149 bp
attB2
NOS
Ubi promoter
Exon
BAR
NOS
Exon
Ubi promoter
P(BLA)
APr

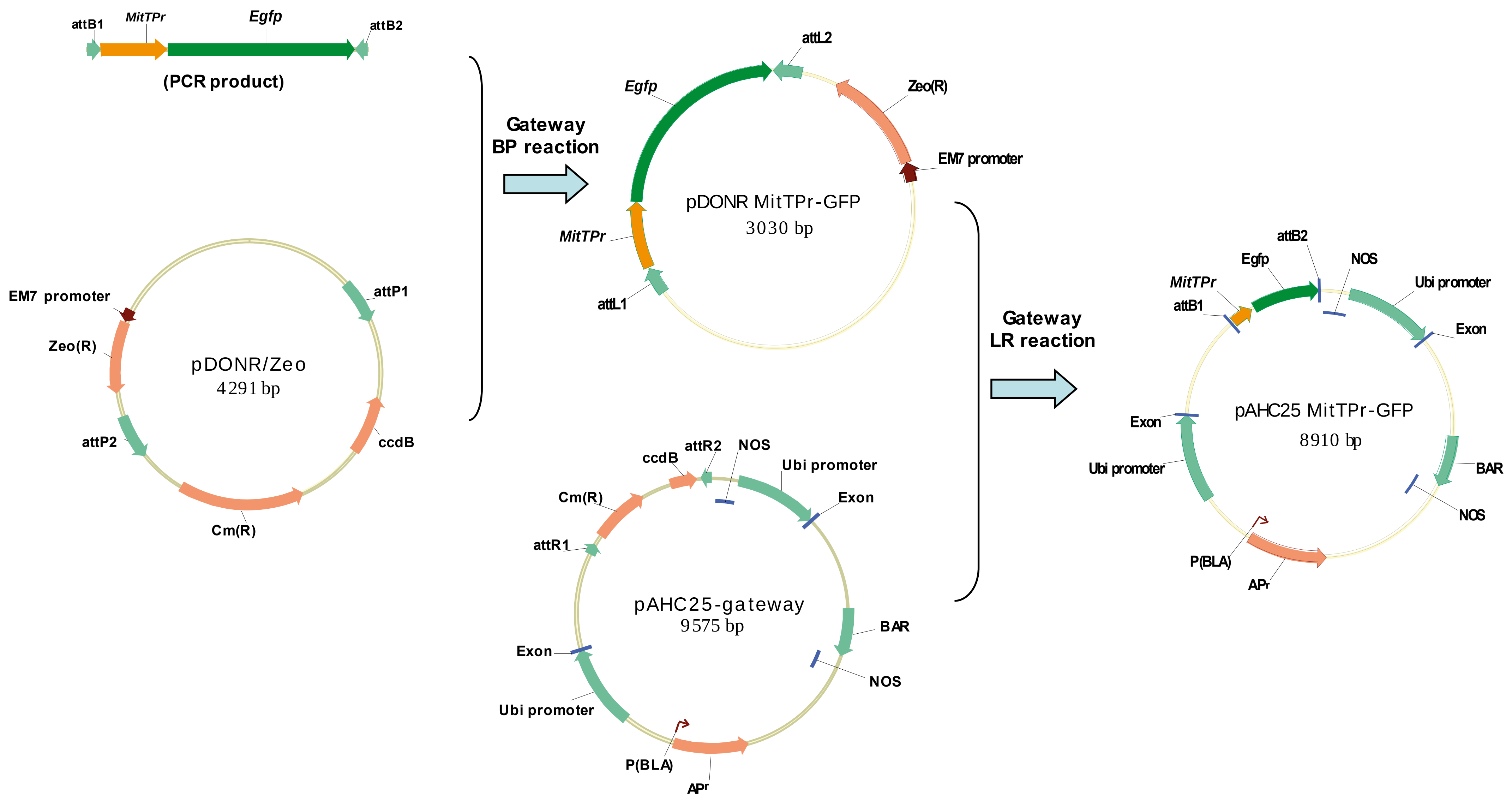
attB1
MitTPr
Egfp
attB2
(PCR product)
EM7 promoter
attP1
Zeo(R)
pDONR/Zeo
4291 bp
attP2
ccdB
Cm(R)
Gateway
BP reaction
attL2
Egfp
Zeo(R)
EM7 promoter
MitTPr
pDONR MitTPr-GFP
3030 bp
attL1
ccdB
attR2
NOS
Ubi promoter
Cm(R)
Exon
attR1
pAHC25-gateway
9575 bp
BAR
Exon
NOS
Ubi promoter
P(BLA)
APr
Gateway
LR reaction
attB2
Egfp
NOS
MitTPr
Ubi promoter
attB1
Exon
Exon
pAHC25 MitTPr-GFP
8910 bp
Ubi promoter
BAR
NOS
P(BLA)
APr
