# Supplementary material for: Mitochondrial Zea mays Brittle1-1 Is a Major Determinant of the Metabolic Fate of Incoming Sucrose and Mitochondrial Function in Developing Maize Endosperms
Source: Front Plant Sci. 2019 Mar 12;10:242. doi: 10.3389/fpls.2019.00242 (PMC6423154; doi:10.3389/fpls.2019.00242)
Supplement: Supplementary file 12 [file Image_4.pdf]

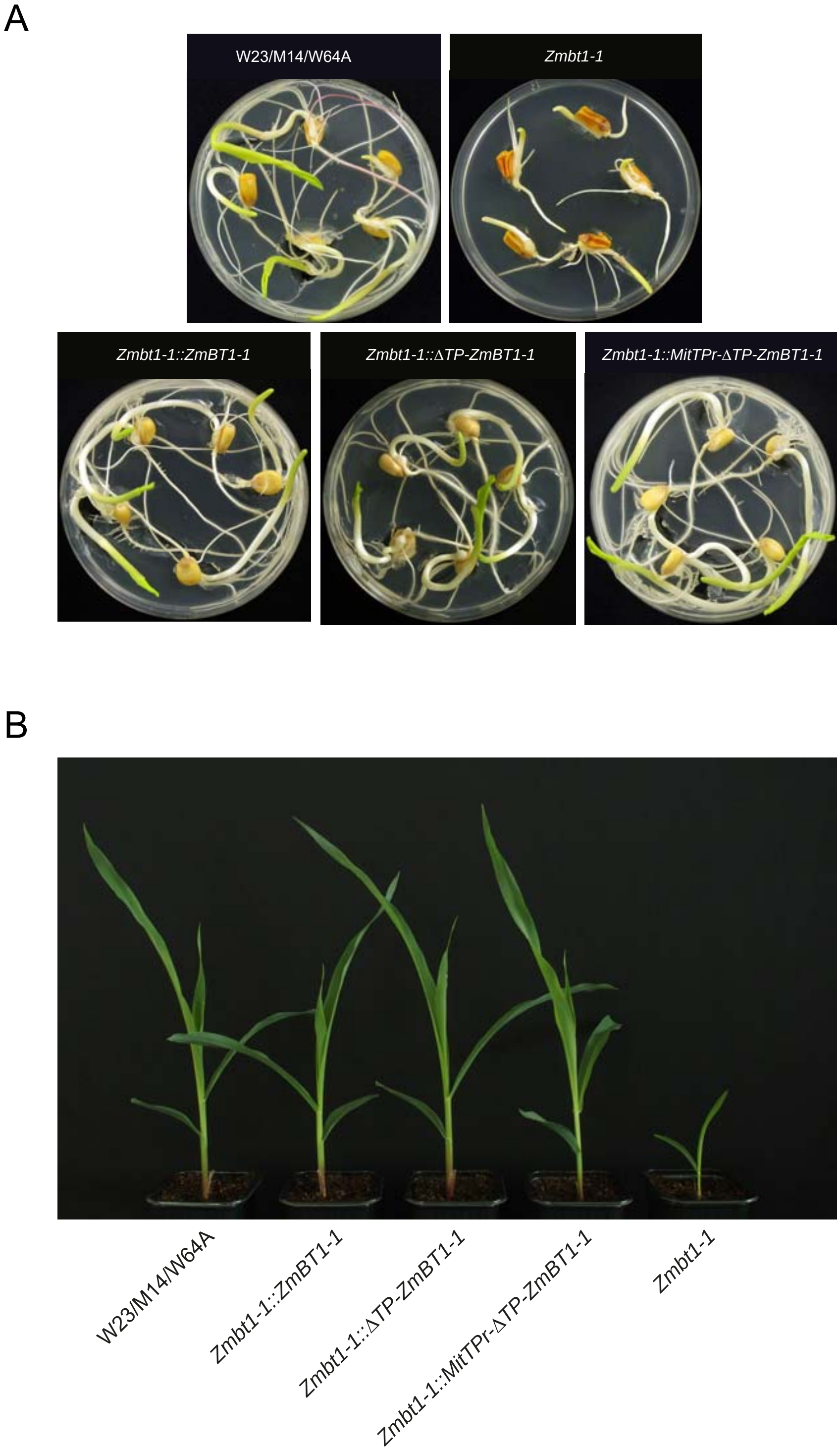

Supplemental Figure 4: Delivery of ZmBT1-1 specifically to mitochondria complements the slow germination and delayed growth phenotype of *Zmbt1-1* plants. Photographs illustrate the morphology of the indicated seedlings and plantlets at 7 and 14 days after sowing (**A** and **B**, respectively).
