# Supplementary material for: Mitochondrial Zea mays Brittle1-1 Is a Major Determinant of the Metabolic Fate of Incoming Sucrose and Mitochondrial Function in Developing Maize Endosperms
Source: Front Plant Sci. 2019 Mar 12;10:242. doi: 10.3389/fpls.2019.00242 (PMC6423154; doi:10.3389/fpls.2019.00242)
Supplement: Supplementary file 13 [file Image_5.pdf]

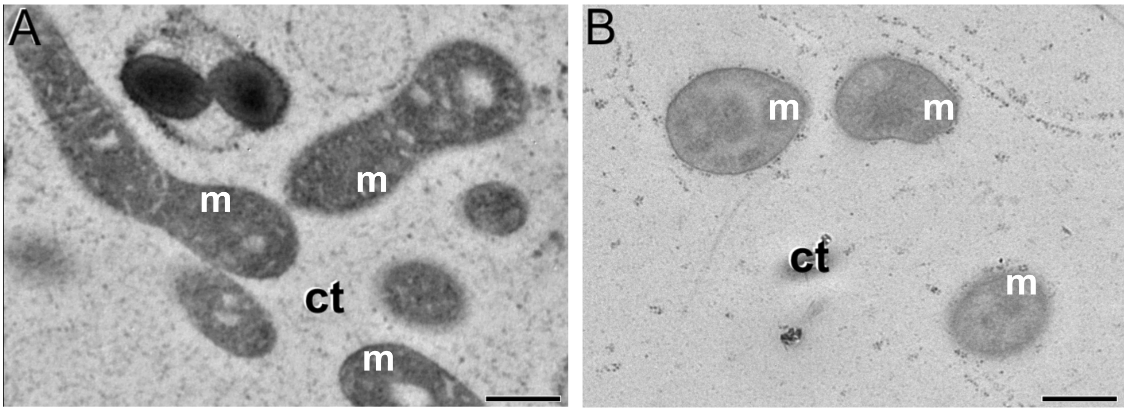


**Supplemental Figure 5:** Ultrastructure of mitochondria (m) in endosperm cells of **(A)** WT and **(B)** *Zmbt1-1* 24 DAP seeds. ct, cytoplasm. Bars: 500 nm.
